# MudPIT Profiling Reveals a Link between Anaerobic Metabolism and the Alkaline Adaptive Response of *Listeria monocytogenes* EGD-e

**DOI:** 10.1371/journal.pone.0054157

**Published:** 2013-01-14

**Authors:** Rolf E. Nilsson, Tom Ross, John P. Bowman, Margaret L. Britz

**Affiliations:** Food Safety Centre, Tasmanian Institute of Agriculture, School of Agricultural Science, University of Tasmania, Hobart, Tasmania, Australia; University of Iowa Carver College of Medicine, United States of America

## Abstract

*Listeria monocytogenes* is a foodborne human pathogen capable of causing life-threatening disease in susceptible populations. Previous proteomic analysis we performed demonstrated that different strains of *L. monocytogenes* initiate a stringent response when subjected to alkaline growth conditions. Here, using multidimensional protein identification technology (MudPIT), we show that in *L. monocytogenes* EGD-e this response involves an energy shift to anaerobic pathways in response to the extracellular pH environment. Importantly we show that this supports a reduction in relative lag time following an abrupt transition to low oxygen tension culture conditions. This has important implications for the packaging of fresh and ready-to-eat foods under reduced oxygen conditions in environments where potential exists for alkaline adaptation.

## Introduction

*Listeria monocytogenes* is a physiologically robust food-borne human pathogen. It is a facultative anaerobe, growing preferentially under microaerophilic conditions. During aerobic growth, energy generation in *L. monocytogenes* is achieved by both fermentation and aerobic respiration. Fermentation is homofermentative and is driven by substrate level phosphorylation (Embden-Meyerhof pathway). *L. monocytogenes* has a split citrate-cycle apparently incapable of energy generation [1], [2]. Aerobic respiration is characterised by the chemiosmotic movement of protons via ATP synthase as the final enzyme of an oxidative phosphorylation pathway [3], [4]. The electron transport chain facilitating oxidative phosphorylation in *L. monocytogenes* is not fully defined, however a cytochrome has been characterised [5], [6]. Under oxygen limited conditions, *L. monocytogenes* is able to generate energy by substrate-level phosphorylation alone (i.e. generation of ATP independent to electron acceptors or cellular respiration) and modulation of its energy generation source (i.e. oxidative versus substrate level phosphorylation) in response to growth conditions has been described (e.g. nutrient limitation) and appears to influence pathogenicity [4], [7], [8]. Oxygen depletion is commonly used for extending the shelf life of packaged fresh and ready-to-eat food products. The ability of *L. monocytogenes* to grow at low oxygen tensions represents a risk for fresh and ready-to-eat food manufacturers, particularly given its association with pathogenicity (e.g. [4]).

*L. monocytogenes* can survive in alkaline conditions up to pH 12, and can grow up to pH 9.5 [9]. Previously, we demonstrated that different strains of *L. monocytogenes* initiate a common stress proteome when subjected to alkaline growth conditions, and that this involves a shift to a survival or "stringent-response"-like state that was coupled to cell surface perturbations which could also aid in attachment to surfaces [10], [11]. In this study we used multidimensional protein identification technology (MudPIT; nano-flow two-dimensional liquid chromatography separation coupled to electrospray tandem mass spectrometry) [12] to detect differential protein expression in alkaline grown *L. monocytogenes* strain EGD-e. Data from these experiments suggested that *L. monocytogenes* strain EGD-e can modulate its source of energy generation following prolonged exposure to elevated concentrations of extracellular hydroxyl ions. This was tested by uncoupling oxidative phosphorylation using an ionophore. A working hypothesis was developed that alkaline grown *L. monocytogenes* strain EGD-e would make the physiological adjustments necessary for transition from aerobic to anaerobic growth and, consequently, would show decreased lag times if subsequently challenged by an abrupt shift to low oxygen tension. This could have important implications for the packaging of fresh and ready-to-eat foods under reduced oxygen conditions.

## Materials and Methods

### Bacterial Strain and Adaptation to Alkaline Culture Conditions

*L. monocytogenes* strain ATCC BAA-679 (EGD-e) was recovered from frozen (−80°C) storage (Protect microbial preservation system; OXOID, Australia) and grown in 10 mL of Tris-buffered brain-heart infusion broth (CM225, ‘BHI’; OXOID, Australia), pH 7.3, incubated aerobically with shaking (50 rpm) at 37°C for twenty hours. The strain was subcultured into fresh Tris-buffered BHI (pH 7.3), incubated as previously described, and the resulting starter culture used to inoculate subsequent cultures. Fresh 9.9 mL Tris-buffered BHI broths were prepared where the pH was adjusted to 7.3 or 9.0 (±0.2) through addition of 4 M NaOH (Sigma-Aldrich, Castle Hill, Australia). After autoclaving, the pH of both media (two×pH7.3, and two×pH9.0) was confirmed using an Orion 250A pH meter (Orion Research Inc, USA), and further adjusted using sterile NaOH or HCl if required. A 100 µL aliquot of the starter culture was transferred to the fresh broths and grown to exponential phase (OD_600_ ≈0.4) aerobically with shaking at 37°C. 100 µL aliquots of these were transferred to fresh 9.9 mL BHI broths (with pH adjusted accordingly) and again incubated aerobically with shaking at 37°C. This was repeated three times to acclimatise the cultures to the growth conditions. The final pH for the pH 7.3 and 9.0 cultures was 7.1 and 8.9 respectively.

### MudPIT Analysis

**Figure 1:**
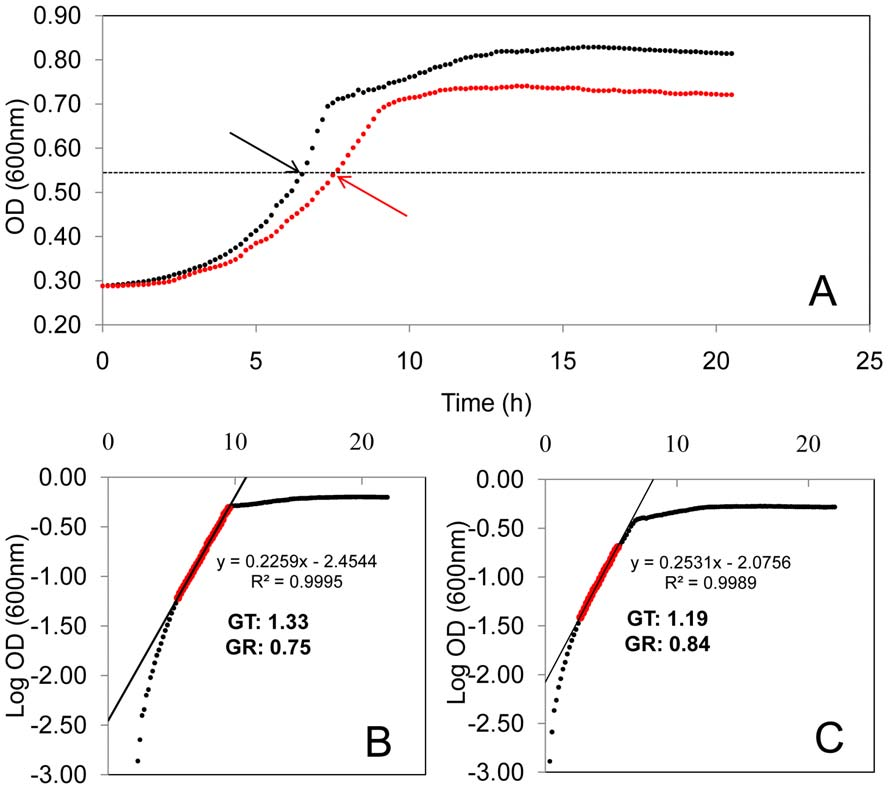
Growth characteristics of *L. monocytogenes* EGD-e cultured aerobically at pH7.3 and 9.0. *L. monocytogenes* EGD-e was cultured in Tris-buffered BHI at pH7.3 (black dots) and 9.0 (red dots) (**A**). Analysis of the growth curves, including calculation of growth rates (pH9.0, **B**, and pH7.3, **C**), was performed according to the method described by Mellefont et al. [20]. The region of the curves used to calculate growth rate are indicated in red. Sampling points for proteomic analysis corresponded to an OD of approximately 0.55 for each treatment and are indicated (arrows).

MudPIT was used to compare the protein expression profile of *L. monocytogenes* strain EGD-e following adaptation to growth at pH9.0 (±0.2). Replicate 10 mL pH7.3 and 9.0 adapted cultures were prepared, incubated at 37°C, and harvested at late exponential phase (OD_600_ ≈0.5–0.6; Figure 1) for proteomic analysis. The cultures were centrifuged at 10,000×*g* for 10 min at 4°C and the supernatant was discarded. The pellets were resuspended in 500 µL of phosphate buffered saline (PBS; pH7.3 and pH9.0±0.2 respectively) and transferred into 1.5 mL Eppendorf Protein Lobind microcentrifuge tubes (Sigma-Aldrich, Castle Hill, NSW, Australia). The tubes were centrifuged at 14,000×*g* for 5 min at 4°C and the PBS supernatant was discarded. The PBS wash was repeated twice. The cell pellets were frozen using liquid nitrogen then thawed on ice for ≈15 min. Soluble proteins were extracted from the cell pellets using a Qproteome bacterial protein preparation kit (37900; Qiagen Pty. Ltd., Victoria, Australia) and approximate concentrations of the protein extracts was determined using a Pierce BCA Protein Assay kit (ThermoFisher Scientific, Victoria, Australia) according to manufacturer instructions.

Volumes of protein extract containing ≈50 µg of protein were transferred to clean Lobind microcentrifuge tubes, frozen with liquid nitrogen, and freeze-dried for ≈8 h using a Dynavac mini ultra-cold vacuum freeze drier (Technolab, Kingston, Tasmania, Australia). The concentrated protein samples were digested with porcine trypsin (Sigma-Aldrich, Castle Hill, NSW, Australia) as described previously [13]. After digestion, the samples were centrifuged at 14000×*g* for 5 min to remove insoluble material and 150 µL of the supernatants were transferred to high pressure liquid chromatography vials (Waters, USA).

MudPIT analysis was performed according to the method described by Delahunty and Yates (2005) using a ThermoFinnegan LTQ Orbitrap tandem mass spectrometer with a nano-electrospray ion source operated with a fragment-ion mass tolerance of 0.5 Daltons. Proteins in the sample were identified by matching the peptides predicted from the tandem mass spectra data against the complete *L. monocytogenes* non-redundant database of the National Centre for Biotechnology Institute (NCBI) using the Computational Proteomics Analysis System (CPAS) Version 8.1 (www.labkey.org). Searches were semi-tryptic, with fixed modifications (cysteine carbamidomethylation-57 Daltons) allowing no missed cleavages, and used the X!Tandem algorithm (www.thegpm.org/tandem/). Spectra counts within each sample were determined using TPP Xpress Quantitation software (Version 2.1) in conjunction with X!Tandem. Functional assignment of protein identifications was predicted manually using The Institute for Genomic Research Comprehensive Microbial Resource (JCVI-CMR) (http://cmr.jcvi.org/tigr-scripts/CMR/GenomePage.cgi?org=ntlm01), GenoList *L. monocytogenes* serovar 1/2a EGD-e database (Version 3) (http://genodb.pasteur.fr/cgi-bin/WebObjects/GenoList.woa/wa/goToTaxoRank?level=Listeria%monocytogenes%20EGD-e), and the Kyoto Encyclopedia of Genes and Genomes (KEGG) (http://www.genome.jp/kegg/).

All searches were run through the Trans Proteomic Pipeline (TPP; Version 3.4) for statistical purposes. The TPP analysis utilised the “Peptide Prophet” and “Protein Prophet” algorithms as previously described [14] to enable the level of false positive peptide and protein identifications to be estimated and to generate a peptide and protein error rate. Identifications with an average peptide prophet error rate (APPER) and protein error rate (PER) of >0.3/1, and protein identifications assigned based on a single unique peptide, were not considered for further analysis. Relative protein abundances between growth conditions were determined using the spectra counting method [15]. Spectra counts were averaged between biological replicates and normalised to account for sampling depth [16]. Statistical significance of differences in spectra abundances for protein identifications between samples was assessed using a likelihood ratio test for independence (*G*-test) adjusted using the William’s correction (*G*_adj_) to reduce false positive rates [17], [18]. Significance was assigned at p≤0.05 (*G*_adj_ ≥3.841). Only those protein identifications that met the filtering criteria (APPER and PER of >0.3/1) and that differed significantly from the control treatment are discussed.

### Uncoupling of Oxidative Phosphorylation

Oxidative phosphorylation was uncoupled in alkaline adapted *L. monocytogenes* EGD-e cells using the ionophore carbonyl cyanide *m*-chlorophenyl hydrazone (CCCP; Sigma-Aldrich, Australia) [6], [19]. Cultures were adapted to growth at pH7.3 and 9.0 as described previously. Replicate 10 mL cultures of each pH condition were prepared, incubated at 37°C, and CCCP was added to give a final concentration of 5 uM at mid-exponential growth phase (OD ≈0.4). Growth was measured turbidimetrically at 600 nm using a Spectronic 20D spectrophotometer (Milton Roy, USA) until the optical density ceased to change.

### Lag phase Determination Following an Abrupt Shift to Low Oxygen Tension

Low oxygen tension culture conditions were prepared using 500 mL of BHI broth in a jacketed New Brunswick BioFlo/CelliGen 115 benchtop fermentor/bioreactor (John Morris Scientific, Australia). A dissolved oxygen (DO) concentration of ≈ 1% (±0.5), measured automatically using a New Brunswick Scientific polarographic DO probe (John Morris Scientific, Australia), was achieved by sparging N_2_ gas into the BHI media, and was maintained by a continual addition of N_2_ at a flow rate of 0.05 L min^−1^ prior to inoculation and during culture. pH was adjusted to 7.3 and 9.0 (±0.1) using sterile 5N NaOH and, if needed, 1N HCl. The cultures were grown at 37°C and were agitated at 50 revolutions per minute using an inbuilt motorised impeller. Replicate 250 mL Tris-buffered BHI cultures of *L. monocytogenes* EGD-e were prepared, adapted to growth at pH7.3 and 9.0 as previously described and grown aerobically with shaking at 37°C. Following adaptation, an aliquot was collected at late exponential phase (OD ≈0.5–0.6) and was used to inoculate replicate microaerobic and aerobic BHI broths (two×pH7.3 and two×pH9.0 for each atmospheric condition) by injection through unidirectional rubber inoculation ports on the fermentors to give a starting OD of 0.3 (±0.05). Growth was monitored by viable counting and the relative lag time of the cultures was determined as described by Mellefont et al. [20].

## Results and Discussion

### Proteomic Analysis of Alkaline Adapted *L. monocytogenes* EGD-e

*L. monocytogenes* EGD-e had a growth rate of 0.75 h^−1^ when cultured aerobically at pH 9.0 compared to 0.84 h^−1^ in the control system (pH7.3) and attained a mean maximum cell density of 9.6×10^7^ CFU mL^−1^ and 6.7×10^9^ CFU mL^−1^ at pH9.0 and 7.3 respectively (Figure 1). A total of 1043 proteins were identified by MudPIT (approximately 37% of the *L. monocytogenes* EGD-e proteome). Application of the filtering criteria (average peptide prophet error rate (APPER) and protein error rate (PER) ≤0.3, unique peptides >1) reduced this to 534 high-confidence protein identifications with an average APPER of 0.096 and 0.085 and an average PER of 0.016 and 0.027 for the pH7.3 and 9.0 growth conditions respectively. These were assigned to functional categories based on the JCVI-CMR *L. monocytogenes* EGD-e functional ontology system (http://cmr.jcvi.org/cgi-bin/CMR/shared/RoleList.cgi) (Table S1). Statistical analysis showed that the abundance of 206 proteins differed significantly between treatments (*G*-test, *p*≤0.05; Table S1).

**Figure 2:**
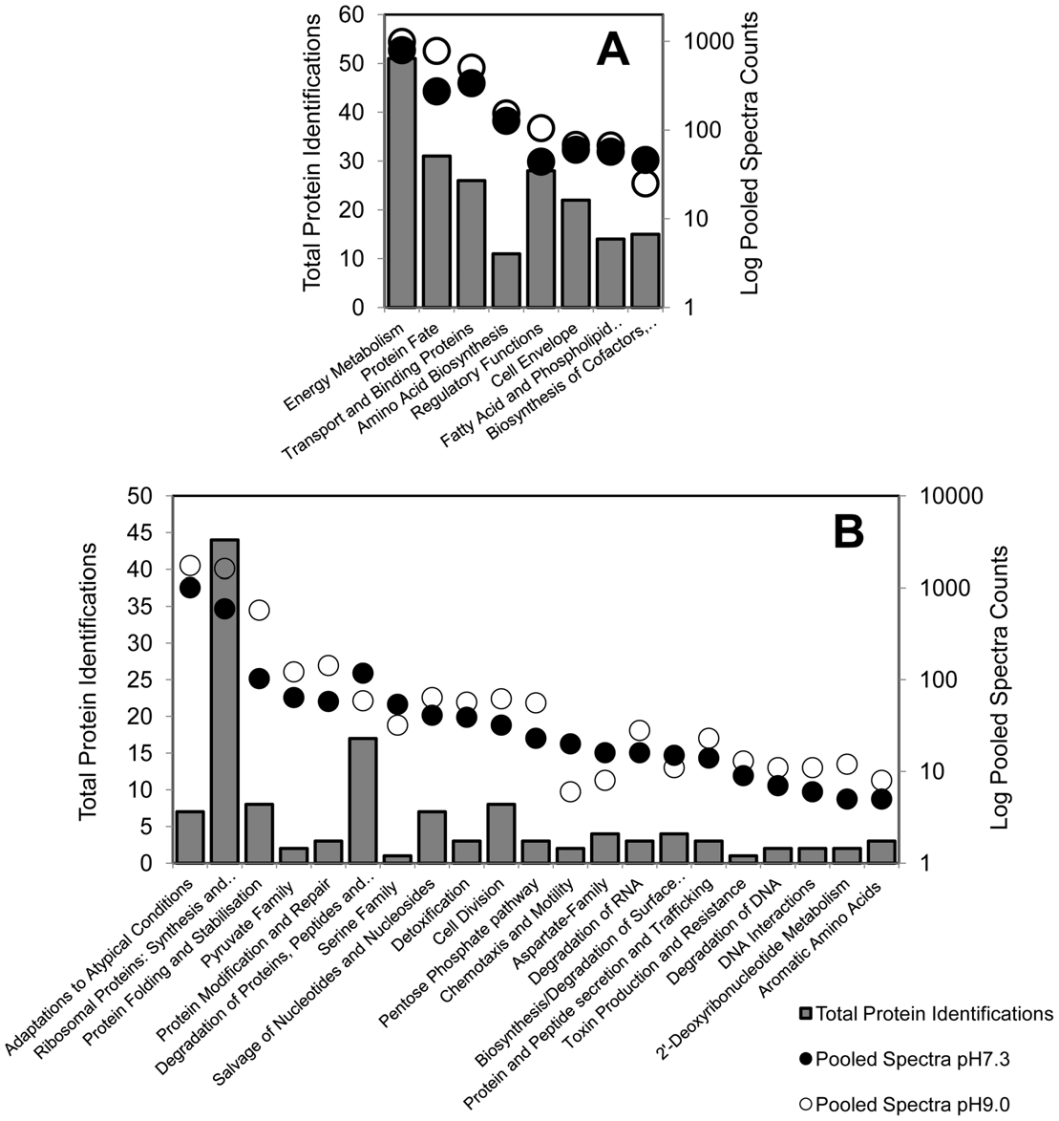
Protein groups identified in the current study previously associated with alkaline pH homeostasis [**6**], [11], [21], [26], [34], [35], [36], [37], [38]. Broad (A) and specific (B) functional grouping categories based on the JCVI-CMR *L. monocytogenes* EGD-e functional ontology system (http://cmr.jcvi.org/cgibin/CMR/shared/RoleList.cgi).

**Figure 3:**
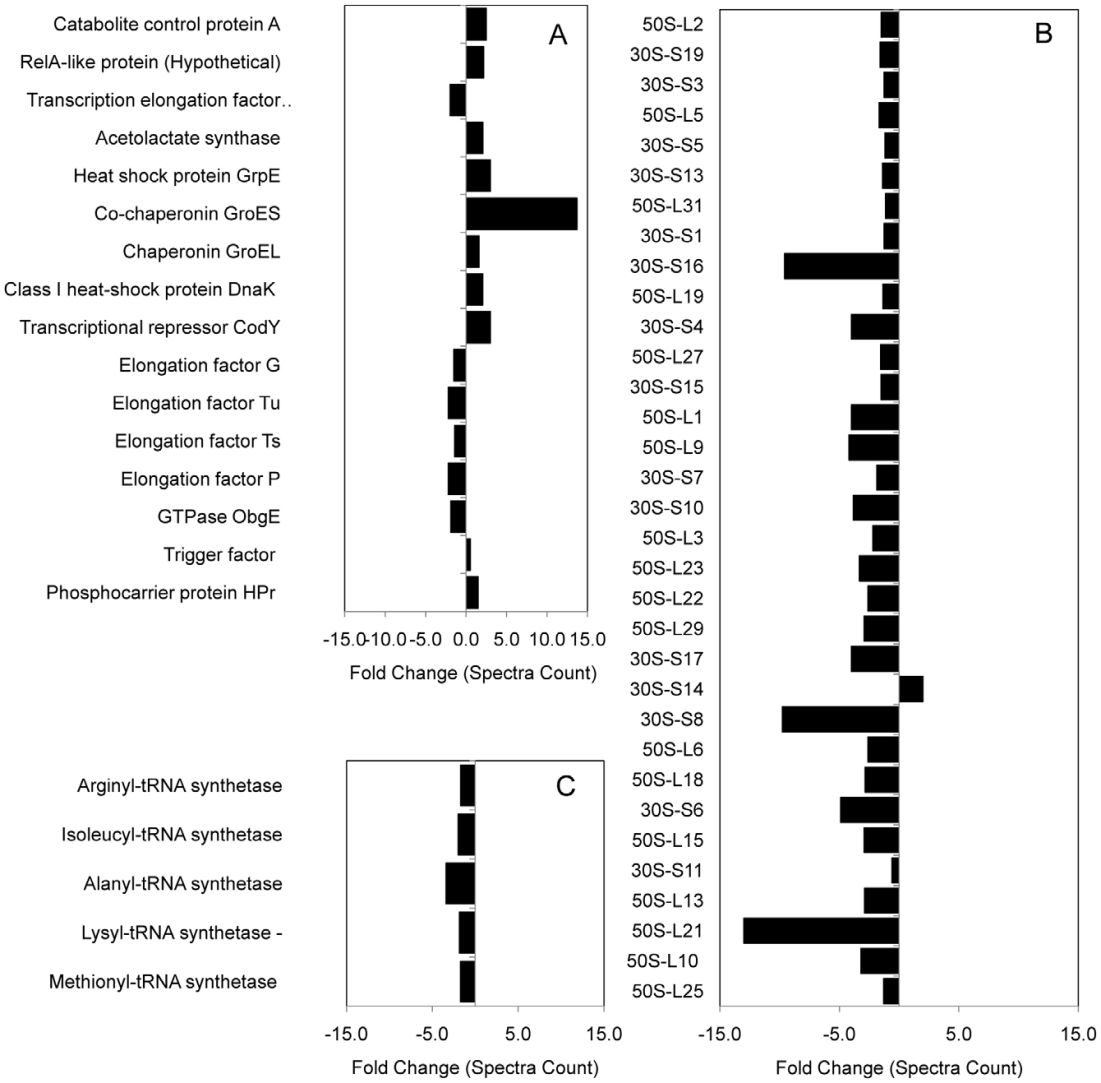
Evidence of stringent response (SR) induction in *L. monocytogenes* following alkaline adaptation. Characteristic expression of proteins previously associated with the bacterial SR was observed. **A**) Increased expression of the RelA synthase (a key enzyme involved in the bacterial SR), stress-related proteins (e.g. GroES, trigger factor and others), transcriptional repressor CodY, acetolactate synthase (AlsS), phosphocarrier protein (HPr), catabolite control protein A (CcpA) and decreased expression of elongation factors (EF’s) and the GTPase ObgE. Increased expression of the RelA synthase initiates production of the SR alarmone guanosine tetraphosphate (ppGpp) [39]. Chaperones stabilise proteins essential for survival during the SR and their expression can reportedly be induced by ObgE protein which is inversely correlated with ppGpp levels and cellular growth rate (as are the EF’s) [40], [41], [42], [43]. Increased expression of AlsS, associated with branched chain amino-acid biosynthesis (BCAAS), reportedly has a role in both pH homeostasis and the bacterial SR representing a shunt of fermentation from end-product generation. Further, BCAAS is regulated by CcpA which, in turn, has been shown to complex with HPr to modulate many genes characteristic of the SR [44], [45]. Similarly, CodY has been shown to be induced as a part of the SR, is associated with regulation of >200 genes and transcriptional repression of CodY is increased in the presence of BCAA’s [42], [46], [47]. **B**) Ribosomal proteins. A global decrease in expression of ribosomal proteins, concordant with a decrease in protein synthesis, is characteristic of the bacterial SR [40], [42], [48]. Further, this decrease in ribosomal proteins correlates with a diminished growth rate and expression of ObgE protein [49]. **C**) Aminoacyl-tRNA synthetase expression is decreased, correlating with the observed decrease in ribosomal proteins and growth rate at alkaline pH [40], [42].

**Figure 4:**
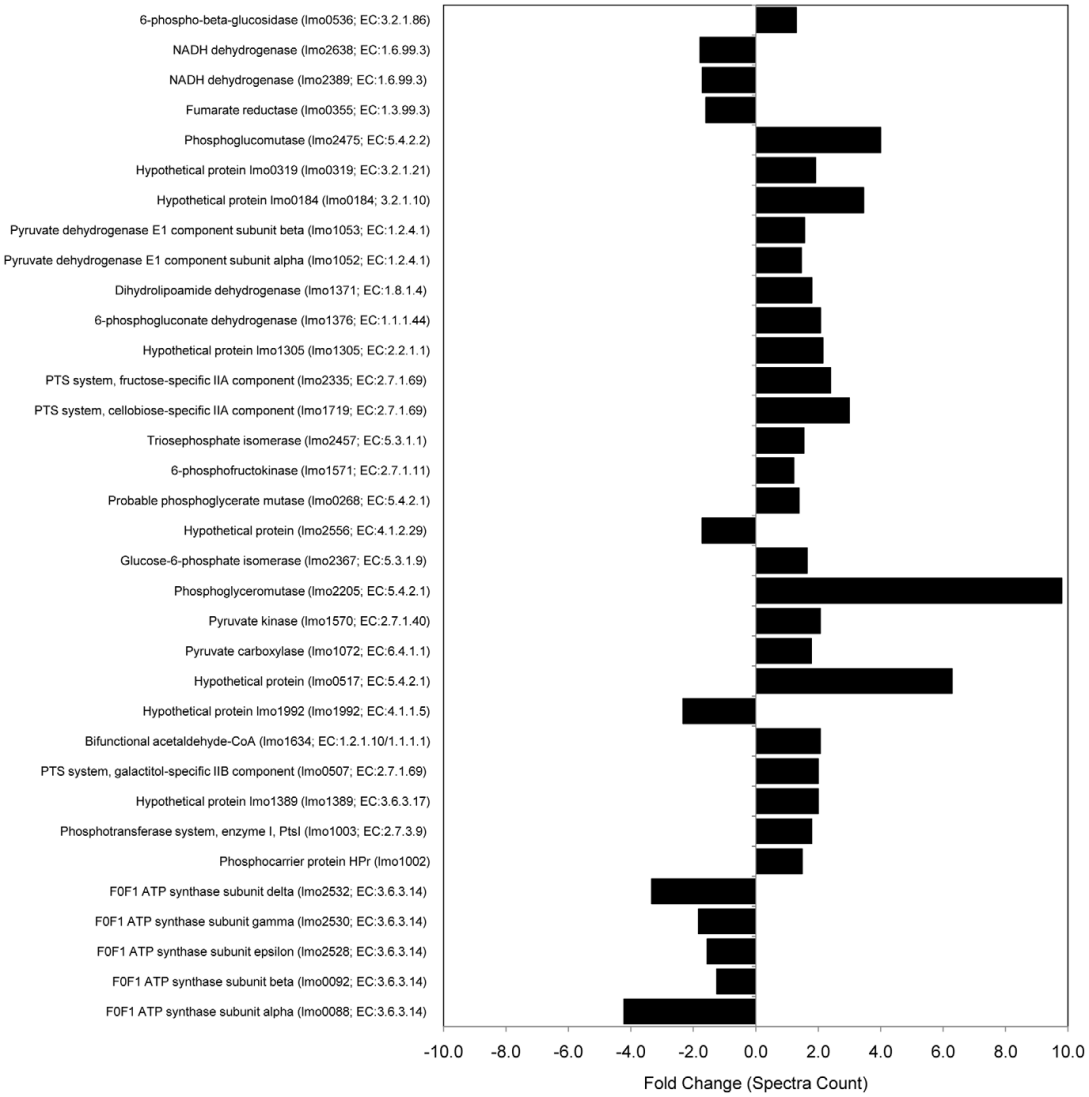
Proteins identified in the current study which are associated with energy metabolism. Fold change (growth at pH9.0 relative to pH7.3) lmo numbers and KEGG (http://www.genome.jp/kegg/) enzyme classification numbers are shown.

**Figure 5:**
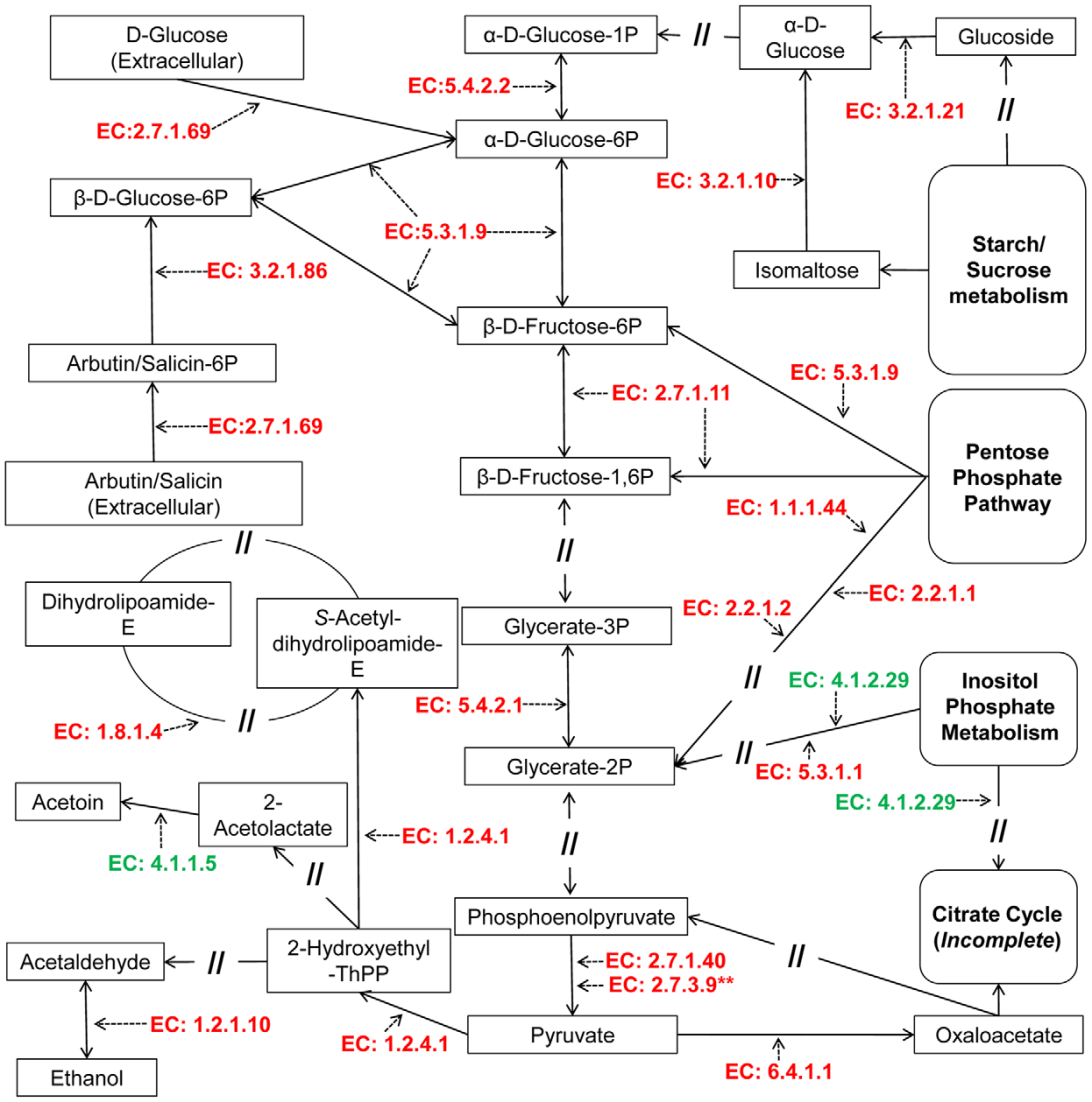
Proteins associated with substrate level phosphorylation observed to be significantly increased (red font = increased, green font = decreased) following adaptation to growth at pH9.0. Sections of pathways where no proteins were identified are indicated with a double forward slash. KEGG enzyme classification numbers are shown. **Transport intermediate.

The protein profile of alkaline adapted *L. monocytogenes* EGD-e agreed with reported observations of pH homeostasis and an alkaline adapted cell physiology (Figure 2). Further, indirect evidence of a stringent-type response was observed and supported previous work investigating the *L. monocytogenes* proteome after adaptation to growth at alkaline pH (Figure 3; [11]). A part of this response involved a shift in energy metabolism from oxidative to substrate level phosphorylation (Figures 4 and 5).

Altering the flow of substrates through these pathways could serve a number of functions under alkaline conditions. The pentose phosphate shunt increases production of reducing equivalents (NADH) directed at fatty acid biosynthesis, the electron transport chain (ETC) and a wide range of other cellular processes. Increased proteins associated with fatty acid biosynthesis and degradation was observed in the present study; however, it was coupled to a decrease in other proteins also associated with biosynthesis of fatty acids (Table S1). While fatty acids are known to have a role in the pH tolerance response of *L. monocytogenes*, it is reported that the type, rather than number, of fatty acid is what imparts the protective effect [21]. On this basis the observed differences in protein abundances associated with fatty acid biosynthesis may reflect the type of fatty acids being produced and/or degraded, however this was not further investigated experimentally.

Importation of sugars via the phosphotransferase system (PTS) is shown to be important for buffering of the cell cytoplasm [22], while increasing substrates for glycolysis. Glycolysis, the pentose phosphate shunt and PTS system produce by-products that are associated with the electron transport chain. This multi-step energy generating system involves a number of protein components that transfer electrons from the initial NADH and succinate donors (generated by the pentose phosphate/glycolysis pathways, and the limited fatty acid degradation observed in the current study), culminating in energy production by an ATP synthase powered by a proton motive force [23]. However, in the present study, decreases in ubiquinone biosynthetic enzymes were detected, along with decreased abundance of proteins associated with ATP-proton motive force (e.g. F0F1 ATPase subunits lmo0092, 0088, 2530, 2532 and 2528) and electron transport chain complexes one, two and five (e.g. NADH dehydrogenase lmo2638 and 2389, and fumarate reductase lmo0355; Figure 4 and 5). A decrease in proton motive force was supported by the increased expression of PTS system proteins (e.g. lmo0507, 1719, 2335, 1002 and 1003; Figure 4). Maintenance of intracellular pH in *L. monocytogenes* was shown by Shabala et al [22] to be coupled to two glucose transport systems: a low-affinity proton motive force-driven system and a high affinity PTS system. As such, should proton motive force be forcibly diminished it could be expected that proteins associated with the PTS-mediated glucose transport system would increase to compensate, as shown in Figure 4.

A diminished ATP-proton motive force would appear to oppose, to some extent, any cytoplasmic acidification process, as the proton pump (driven by the proton motive force) expels protons in the generation of energy (ATP) via an ATP synthase. Considering the decreased proton motive force and associated protein abundances, it is possible that under alkaline conditions the external pH is causing loss of protons, decreasing the flow back through the ATP synthase, and resulting in a net loss of protons from the cytoplasm (a reversal of the ATP synthase reaction). This would lead to a decrease in the proton motive force and could drive a decrease in ATP synthase in order to maximise proton retention within the cytoplasm. A decrease in ATP synthase activity in cells grown at alkaline pH has been demonstrated previously [24]. Consequently, we hypothesised that the mechanism driving the observed decrease in ATP synthase activity in alkaline adapted cells is a diminished proton motive force caused by the high cell surface pH (low proton concentration) resulting from the alkaline culture media. This concept is described in a microbial attachment model introduced by Hong and Brown [25]. In their model, they suggest that a charge-regulation effect may be induced by the environmental pH (attachment surface in their study) in proximity to the cell surface, resulting in a net migration of protons out of the cell. This migration of protons can shift the equilibrium of the ATP synthase phosphorylation reaction [24], [25], [26]. The associated deficit in energy (ATP) production may be offset by the observed increase in substrate level phosphorylation in the present study (Figure 5).

The combination of these mechanisms of acidification, including the charge regulation effect, could ultimately lead to a reducing environment within the cytoplasm, with a subsequent increase in reactive oxygen species through electron leakage [27]. This was suggested in the current study from the observation of significantly increased abundance of lmo1407 (pyruvate formate lyase; Table S1). This protein, generally associated with anaerobic metabolism, has been observed to increase under oxidative stress in the presence of increased reactive oxygen species [28]. Similarly, Listeria adhesion protein (lmo1634) was significantly increased at pH 9.0 and induction of this protein under anaerobic conditions has been described previously [29]. Given the evidence generated from the combined proteomics dataset we proposed that an energy generation shift towards fermentation was occurring during alkaline adaptation.

### Uncoupling of Oxidative Phosphorylation and Relative Lag Time after an Abrupt Shift to Low Oxygen Tension

Proteomic analysis indicated that an energy shift induced in *L. monocytogenes* by prolonged exposure to alkaline culture conditions could support anaerobiosis and involved down-regulation of oxidative phosphorylation. To test whether oxidative phosphorylation was reduced alkaline adapted and non-adapted cells were exposed to carbonyl *m*-chlorophenyl hydrazone (CCCP). CCCP is a chemical inhibitor of oxidative phosphorylation, achieved by uncoupling the proton gradient and consequently, interfering with ATP synthase’s ability to generate ATP [30]. Should alkaline adapted *L. monocytogenes* be more reliant on substrate-level rather than oxidative phosphorylation increased survival when exposed to CCCP would be expected.

**Figure 6:**
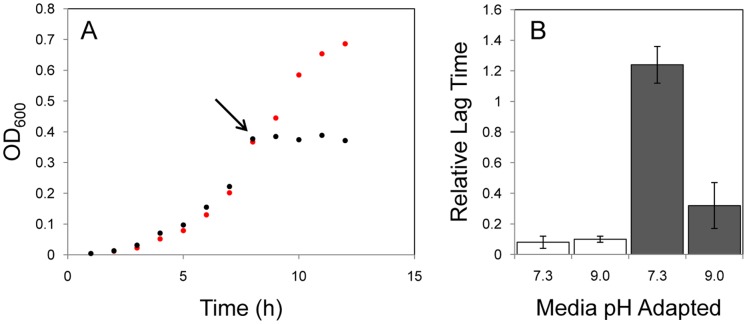
Ionophore and atmospheric challenge after adaptation to growth at pH9.0. (**A**) *L. monocytogenes* EGD-e was adapted to growth at pH7.3 (squares) and pH9.0 (circles) and challenged with 5 uM CCCP. Addition of CCCP is indicated (arrow). **B**) Relative lag time after an abrupt shift to low oxygen tension. Unfilled bars show aerobic culture. Filled bars show 1% (±0.5) oxygen tension.

Addition of CCCP inhibited growth at pH7.3, while growth continued for the pH 9.0 grown cells (Figure 6A). This is consistent with a shift to predominantly substrate-level phosphorylation from oxidative phosphorylation and, when coupled with our proteomic findings, the transition to anaerobiosis. This conclusion was further supported by a significant decrease in expression of acetolactate decarboxylase (lmo1992; Figures 4 and 5), the final enzyme in the acetoin biosynthesis pathway and a metabolic indicator of anaerobic growth in *L. monocytogenes* [31]. Furthermore, acetoin was not detected in culture fluids of alkaline adapted *L. monocytogenes* EGD-e cells using a Voges-Proskauer method [32] but was at pH 7.3 (data not shown).

Importantly, relative lag time (RLT) following an abrupt shift to low oxygen tension (1% ±0.5) supported a transition to anaerobiosis during alkaline adaptation, with reduced RLT for alkaline adapted *L. monocytogenes* EGD-e relative to non-adapted (pH 7.3) (Figure 6B). This is an important finding given that removal of air is a commonly applied food preservation hurdle, and indicates that alkaline adapted *L. monocytogenes* EGD-e may be capable of reaching dangerous numbers under such conditions faster than non-alkaline adapted cells.

### Conclusions

MudPIT analysis indicated that alkaline pH homeostasis in *L. monocytogenes* EGD-e results from multiple regulatory mechanisms driven by the necessity to increase cytoplasmic acidity, maintain cellular integrity, and minimise other physical effects imparted by the extracellular environment. A key component of this response involves direct cytoplasmic acidification through the production, retention and importation of polar or charged proteins, peptides and amino acids. Furthermore, systems are mobilised that facilitate stabilisation of these and other regulatory proteins to prevent pH induced conformational changes that may lead to loss of function. This is coupled with an adaptive shift in energy metabolism that increases production of acidic by-products, ATP and reducing equivalents, to compensate for inhibition of other energy production pathways that are physically influenced by the extracellular pH environment. This includes restriction of the electron transport chain and inversion of the proton motive force to conserve protons that are being lost due to a charge-regulation effect, and to increase the pool of oxidised reducing equivalents.

Importantly, the modified physiology of alkaline adapted *L. monocytogenes* matches that expected during anaerobiosis and allows for rapid growth (decreased lag time) following an abrupt shift to low oxygen tension. This is likely to be limited to very low oxygen tensions rather than strict anaerobiosis as increased recruitment of the aerobic Class Ia ribonucleotide reductase (RNR) system (e.g. lmo2155) was evident in the alkaline adapted cells, possibly in an effort to ‘scavenge’ the limited oxygen available, while the abundance of anaerobic Class III RNR (e.g. lmo2079) while increased, did not differ significantly. Class III RNR is essential for growth of *L. monocytogenes* under strict anaerobic conditions, and in *L. monocytogenes* EGD-e, this protein is non-functional due to a deletion in a key catalytic site [33]. Results from this work suggest that *L. monocytogenes* EGD-e is able to grow at very low oxygen tensions and may still be a suitable strain for experiments using such conditions.

This has important food safety implications, showing that alkaline adaptation in *L. monocytogenes* is able to generate a phenotype capable of proliferating in very low oxygen tensions faster than non-adapted cells. This has relevance to food packaging procedures that employ the removal of oxygen as a growth limiting hurdle. Consequently, the combination of food packaged under low oxygen tension and the potential for *L. monocytogenes* to become adapted to alkaline agents must be a consideration when assessing the risk of food contaminations by this organism. Further work is needed to investigate how this translates to the nutrient flux (including nutrient limitation) *L. monocytogenes* likely encounters within food processing and production environments.

## Supporting Information

Table S1 Proteins recovered from *L. monocytogenes* EGD-e after adaptation to growth in BHI media with the pH adjusted to 7.3 and 9.0. PER = Protein Error Rate; APPER = Average Peptide Prophet Error Rate; UP = Unique Peptides. Functional assignment of protein identifications was predicted manually using The Institute for Genomic Research Comprehensive Microbial Resource (JCVI-CMR) (http://cmr.jcvi.org/tigr-scripts/CMR/GenomePage.cgi?org=ntlm01). Significantly different protein abundances (*G*-test; p≤0.05) are indicated with shading and their common names are shown.
(DOC)

## References

[1] Trivett TL (1971) Citrate cycle and related metabolism of *Listeria monocytogenes* . J Bacteriol 107: 770–777. 4999414 10.1128/jb.107.3.770-779.1971 PMC246999

[2] Eisenreich W, Slaghuis J, Laupitz R, Bussemer J, Stritzker J, et al (2006) C-13 isotopologue perturbation studies of *Listeria monocytogenes* carbon metabolism and its modulation by the virulence regulator PrfA. PNAS 103: 2040–2045. 16461909 10.1073/pnas.0507580103 PMC1413697

[3] Daneshvar MI, Brooks JB, Malcolm GB, Pine L (1989) Analysis of fermentation products of Listeria species by frequency-pulsed electron-capture gas-liquid chromatography. Can J Microbiol 35: 786–793. 2510917 10.1139/m89-131

[4] Lungu B, Ricke SC, Johnson MG (2009) Growth, survival, proliferation and pathogenesis of *Listeria monocytogenes* under low oxygen or anaerobic conditions: A review. Anaerobe 15: 7–17. 18926916 10.1016/j.anaerobe.2008.08.001

[5] Sleator RD, Gahan CGM, Hill C (2003) A postgenomic appraisal of osmotolerance in *Listeria monocytogenes* . Appl Environ Microbiol 69: 1–9. 12513970 10.1128/AEM.69.1.1-9.2003 PMC152475

[6] Chaturongakul S, Boor KJ (2006) Sigma(B) activation under environmental and energy stress conditions in *Listeria monocytogenes* . Appl Environ Microbiol 72: 5197–5203. 16885265 10.1128/AEM.03058-05 PMC1538764

[7] Ebersbach T, Jorgensen JB, Heegaard PM, Lahtinen SJ, Ouwehand AC, et al (2010) Certain dietary carbohydrates promote Listeria infection in a guinea pig model, while others prevent it. Int J Food Microbiol 140: 218–224. 20417983 10.1016/j.ijfoodmicro.2010.03.030

[8] Lungu B, Saldivar JC, Story R, Ricke SC, Johnson MG (2010) The combination of energy-dependent internal adaptation mechanisms and external factors enables *Listeria monocytogenes* to express a strong starvation survival response during multiple-nutrient starvation. Foodborne Pathog Dis 7: 499–505. 20001327 10.1089/fpd.2009.0408

[9] Tienungoon S, Ratkowsky DA, McMeekin TA, Ross T (2000) Growth limits of *Listeria monocytogenes* as a function of temperature, pH, NaCl, and lactic acid. Appl Environ Microbiol 66: 4979–4988. 11055952 10.1128/aem.66.11.4979-4987.2000 PMC92408

[10] Nilsson RE, Ross T, Bowman JP (2011) Variability in biofilm production by *Listeria monocytogenes* correlated to strain origin and growth conditions. Int J Food Microbiol 150: 14–24. 21824672 10.1016/j.ijfoodmicro.2011.07.012

[11] Nilsson RE, Latham R, Mellefont L, Ross T, Bowman JP (2012) MudPIT analysis of alkaline tolerance by *Listeria monocytogenes* strains recovered as persistent food factory contaminants. Food Microbiol 30: 187–196. 22265300 10.1016/j.fm.2011.10.004

[12] Delahunty CM, Yates JR III (2007) MudPIT: multidimensional protein identification technology. Biotech 43: 563–569. 18072585

[13] Delahunty C, Yates JR (2005) Protein identification using 2D-LC-MS/MS. Methods 35: 248–255. 15722221 10.1016/j.ymeth.2004.08.016

[14] Keller A, Nesvizhskii AI, Kolker E, Aebersold R (2002) Empirical statistical model to estimate the accuracy of peptide identifications made by MS/MS and database search. Anal Chem 74: 5383–5392. 12403597 10.1021/ac025747h

[15] Wang WX, Zhou HH, Lin H, Roy S, Shaler TA, et al (2003) Quantification of proteins and metabolites by mass spectrometry without isotopic labeling or spiked standards. Anal Chem 75: 4818–4826. 14674459 10.1021/ac026468x

[16] Beissbarth T, Hyde L, Smyth GK, Job C, Boon W-M, et al (2004) Statistical modeling of sequencing errors in SAGE libraries. Bioinformatics 20 Suppl 1i31–39. 15262778 10.1093/bioinformatics/bth924

[17] Sokal R, Rohlf F (1995) Biometry: The principles and practice of statistics in biological research. New York: W.H. Freeman.

[18] Zhang B, VerBerkmoes NC, Langston MA, Uberbacher E, Hettich RL, et al (2006) Detecting differential and correlated protein expression in label-free shotgun proteomics. J Prot Res 5: 2909–2918. 10.1021/pr0600273 17081042

[19] Gill AO, Holley RA (2004) Mechanisms of bactericidal action of cinnamaldehyde against *Listeria monocytogenes* and of eugenol against *L. monocytogenes* and *Lactobacillus sakei* . Appl Environ Microbiol 70: 5750–5755. 15466510 10.1128/AEM.70.10.5750-5755.2004 PMC522076

[20] Mellefont LA, McMeekin TA, Ross T (2003) The effect of abrupt osmotic shifts on the lag phase duration of foodborne bacteria. Int J Food Microbiol 83: 281–293. 12745233 10.1016/s0168-1605(02)00377-x

[21] Giotis ES, McDowell DA, Blair IS, Wilkinson BJ (2007) Role of branched-chain fatty acids in pH stress tolerance in *Listeria monocytogenes* . Appl Environ Microbiol 73: 997–1001. 17114323 10.1128/AEM.00865-06 PMC1800763

[22] Shabala L, Budde B, Ross T, Siegumfeldt H, McMeekin T (2002) Responses of *Listeria monocytogenes* to acid stress and glucose availability monitored by measurements of intracellular pH and viable counts. Int J Food Microbiol 75: 89–97. 11999120 10.1016/s0168-1605(01)00740-1

[23] Alberts B, Bray D, Hopkin K, Johnson A, Lewis J, et al. (2004) Protein structure and function. In: Alberts B, Bray D, Hopkin K, Johnson A, Lewis J et al.., editors. Essential Cell Biology. New York: Garland Science. 119–167.

[24] von Ballmoos C, Cook GM, Dimroth P (2008) Unique rotary ATP synthase and its biological diversity. Annual Review of Biophysics. 43–64. 10.1146/annurev.biophys.37.032807.130018 18573072

[25] Hong Y, Brown DG (2010) Alteration of bacterial surface electrostatic potential and pH upon adhesion to a solid Surface and impacts to cellular bioenergetics. Biotech Bioeng 105: 965–972. 10.1002/bit.22606 19953670

[26] Booth IR (1985) Regulation of cytoplasmic pH in bacteria. Microbiol Rev 49: 359–378. 3912654 10.1128/mr.49.4.359-378.1985 PMC373043

[27] Schafer FQ, Buettner GR (2001) Redox environment of the cell as viewed through the redox state of the glutathione disulfide/glutathione couple. Free Radic Biol Med 30: 1191–1212. 11368918 10.1016/s0891-5849(01)00480-4

[28] Feng H, Xiang H, Zhang J, Liu G, Guo N, et al. (2009) Genome-Wide Transcriptional Profiling of the Response of *Staphylococcus aureus* to Cryptotanshinone. J Biomed Biotech: DOI:61750910.61751155/61752009/61617509. 10.1155/2009/617509 PMC2730559 19707532

[29] Burkholder KM, Kim K-P, Mishra KK, Medina S, Hahm B-K, et al (2009) Expression of LAP, a SecA2-dependent secretory protein, is induced under anaerobic environment. Microbes Infect 11: 859–867. 19454322 10.1016/j.micinf.2009.05.006

[30] Ito M, Ohnishi Y, Itoh S, Nishimura M (1983) Carbonyl cyanide-*m-*chlorophenyl hydrazone-rsistant *Escherichia coli* mutant that exhibits a temperature-senitive unc phenotype J Bacteriol. 153: 310–315. 10.1128/jb.153.1.310-315.1983 PMC217372 6217194

[31] Romick TL, Fleming HP, McFeeters RF (1996) Aerobic and anaerobic metabolism of *Listeria monocytogenes* in defined glucose medium. Appl Environ Microbiol 62: 304–307. 8572709 10.1128/aem.62.1.304-307.1996 PMC167799

[32] Barry AL, Feeney KL (1967) 2 quick methods for Voges-Proskauer test Appl Microbiol. 15: 1138–1142. 10.1128/am.15.5.1138-1141.1967 PMC547154 4865027

[33] Ofer A, Kreft J, Logan DT, Cohen G, Borovok I, et al (2011) Implications of the Inability of *Listeria monocytogenes* EGD-e To Grow Anaerobically Due to a Deletion in the Class III NrdD Ribonucleotide Reductase for Its Use as a Model Laboratory Strain. J Bacteriol 193: 2931–2940. 21478338 10.1128/JB.01405-10 PMC3133202

[34] Gardan R, Cossart P, Labadie J, European Listeria Genome C (2003) Identification of *Listeria monocytogenes* genes involved in salt and alkaline-pH tolerance. Appl Environ Microbiol 69: 3137–3143. 12788708 10.1128/AEM.69.6.3137-3143.2003 PMC161542

[35] Giotis ES, Blair IS, McDowell DA (2007) Morphological changes in *Listeria monocytogenes* subjected to sublethal alkaline stress. Int J Food Microbiol 120: 250–258. 17935812 10.1016/j.ijfoodmicro.2007.08.036

[36] Hunte C, Screpanti E, Venturi M, Rimon A, Padan E, et al (2005) Structure of a Na+/H+ antiporter and insights into mechanism of action and regulation by pH. Nature 435: 1197–1202. 15988517 10.1038/nature03692

[37] Lewinson O, Padant E, Bibi E (2004) Alkalitolerance: A biological function for a multidrug transporter in pH homeostasis. PNAS 101: 14073–14078. 15371593 10.1073/pnas.0405375101 PMC521123

[38] Padan E, Bibi E, Ito M, Krulwich TA (2005) Alkaline pH homeostasis in bacteria: New insights. Biochim Biophys Acta Biomem 1717: 67–88. 10.1016/j.bbamem.2005.09.010 PMC3072713 16277975

[39] Avarbock D, Avarbock A, Rubin H (2000) Differential regulation of opposing Rel(Mtb) activities by the aminoacylation state of a tRNA center dot ribosome center dot mRNA center dot Rel(Mtb) complex. Biochem 39: 11640–11648. 10995231 10.1021/bi001256k

[40] Marr AG (1991) Growth-rate of *Escherichia coli* . Microbiol Rev 55: 316–333. 1886524 10.1128/mr.55.2.316-333.1991 PMC372817

[41] Persky NS, Ferullo DJ, Cooper DL, Moore HR, Lovett ST (2009) The ObgE/CgtA GTPase influences the stringent response to amino acid starvation in *Escherichia coli* . Molec Microbiol 73: 253–266. 19555460 10.1111/j.1365-2958.2009.06767.x PMC2771346

[42] Potrykus K, Cashel M (2008) (p)ppGpp: Still Magical? Annual Review of Microbiology. 35–51. 10.1146/annurev.micro.62.081307.162903 18454629

[43] Sato A, Kobayashi G, Hayashi H, Yoshida H, Wada A, et al (2005) The GTP binding protein Obg homolog ObgE is involved in ribosome maturation. Genes Cells 10: 393–408. 15836769 10.1111/j.1365-2443.2005.00851.x

[44] Christensen DP, Benson AK, Hutkins RW (1999) Mutational analysis of the role of HPr in *Listeria monocytogenes* . Appl Environ Microbiol 65: 2112–2115. 10224008 10.1128/aem.65.5.2112-2115.1999 PMC91305

[45] Dalebroux ZD, Svensson SL, Gaynor EC, Swanson MS (2010) ppGpp Conjures Bacterial Virulence. Microbiol Mol Biol Rev 74: 171–174. 20508246 10.1128/MMBR.00046-09 PMC2884408

[46] Handke LD, Shivers RP, Sonenshein AL (2008) Interaction of *Bacillus subtilis* CodY with GTP. J Bacteriol 190: 798–806. 17993518 10.1128/JB.01115-07 PMC2223590

[47] Miethke M, Westers H, Blom E-J, Kuipers OP, Marahiel MA (2006) Iron starvation triggers the stringent response and induces amino acid biosynthesis for bacillibactin production in *Bacillus subtilis* . J Bacteriol 188: 8655–8657. 17012385 10.1128/JB.01049-06 PMC1698241

[48] Svitil AL, Cashel M, Zyskind JW (1993) Guanosine tetraphosphate inhibits protein-synthesis invivo- A possible protective mechanism for starvation stress in *Escherichia coli* . J Biol Chem 268: 2307–2311. 8428905

[49] Bremer H, Dennis P (1996) Modulation of chemical composition and other parameters of the cell by growth rate; Neidhardt F, editor. Washington: ASM Press.

