# Supplementary material for: MudPIT Profiling Reveals a Link between Anaerobic Metabolism and the Alkaline Adaptive Response of Listeria monocytogenes EGD-e
Source: PLoS One. 2013 Jan 14;8(1):e54157. doi: 10.1371/journal.pone.0054157 (PMC3544664; doi:10.1371/journal.pone.0054157)
Supplement: Table S1 — Proteins recovered from L. monocytogenes EGD-e after adaptation to growth in BHI media with the pH adjusted to 7.3 and 9.0. PER = Protein Error Rate; APPER = Average Peptide Prophet Error Rate; UP = Unique Peptides. Functional assignment of protein identifications was predicted manually using The Institute for Genomic Research Comprehensive Microbial Resource (JCVI-CMR) (http://cmr.jcvi.org/tigr-scripts/CMR/GenomePage.cgi?org=ntlm01). Significantly different protein abundances (G-test; p≤0.05) are indicated with shading and their common names are shown. (DOC) [file pone.0054157.s001.doc]

**Table S1.**

| **Protein** | **Locus** | **NCBI GI Code** | **Main-Role** | **Sub-Role** | **Spectra Count** | | **pH7.3** | |  | **pH9.0** | |  |
| --- | --- | --- | --- | --- | --- | --- | --- | --- | --- | --- | --- | --- |
|  |  |  |  |  | **pH7.3** | **pH9.0** | **PER** | **APPER** | **UP** | **PER** | **APPER** | **UP** |
|  | lmo0001 | 16802049 | DNA Metabolism | DNA Replication, Recombination and Repair | 5 | 9 | 0.069 | 0.107 | 2 | 0.092 | 0.027 | 2 |
|  | lmo0002 | 16802050 | DNA Metabolism | DNA Replication, Recombination and Repair | 12 | 10 | 0.000 | 0.000 | 2 | 0.000 | 0.011 | 2 |
|  | lmo0004 | 16802052 | Function Unknown | Unknown General | 7 | 5 | 0.000 | 0.166 | 2 | 0.092 | 0.079 | 2 |
|  | lmo0006 | 16802054 | DNA Metabolism | DNA Replication, Recombination and Repair | 6 | 7 | 0.064 | 0.043 | 2 | 0.101 | 0.205 | 2 |
|  | lmo0007 | 16802055 | DNA Metabolism | DNA Replication, Recombination and Repair | 9 | 7 | 0.000 | 0.140 | 2 | 0.000 | 0.062 | 2 |
|  | lmo0018 | 16802066 | Central Intermediary Metabolism | Other | 12 | 10 | 0.076 | 0.000 | 2 | 0.101 | 0.009 | 2 |
|  | lmo0024 | 16802072 | Multiple Roles | Multiple Roles | 7 | 5 | 0.069 | 0.186 | 2 | 0.092 | 0.196 | 2 |
| hypothetical protein lmo0027 | lmo0027 | 16802075 | Multiple Roles | Multiple Roles | 31 | 131 | 0.000 | 0.017 | 4 | 0.000 | 0.051 | 14 |
| 30S ribosomal protein S6 | lmo0044 | 16802092 | Protein Synthesis | Ribosomal Proteins: Synthesis and Modification | 186 | 38 | 0.000 | 0.042 | 5 | 0.000 | 0.013 | 20 |
|  | lmo0045 | 16802093 | DNA Metabolism | DNA Replication, Recombination and Repair | 12 | 10 | 0.064 | 0.144 | 2 | 0.009 | 0.124 | 2 |
|  | lmo0046 | 16802094 | Protein Synthesis | Ribosomal Proteins: Synthesis and Modification | 7 | 7 | 0.000 | 0.123 | 2 | 0.101 | 0.031 | 2 |
| 50S ribosomal protein L9 | lmo0053 | 16802101 | Protein Synthesis | Ribosomal Proteins: Synthesis and Modification | 21 | 5 | 0.000 | 0.053 | 2 | 0.000 | 0.080 | 3 |
|  | lmo0055 | 16802103 | Purines, Pyrimidines, Nucleosides and Nucleotides | Purine Ribonucleotide Biosynthesis | 8 | 11 | 0.000 | 0.076 | 2 | 0.069 | 0.000 | 2 |
| F0F1 ATP synthase subunit alpha | lmo0088 | 16802136 | Energy Metabolism | ATP Proton Motive Force Interconversion | 38 | 9 | 0.000 | 0.134 | 5 | 0.000 | 0.000 | 3 |
| F0F1 ATP synthase subunit beta | lmo0092 | 16802140 | Energy Metabolism | ATP Proton Motive Force Interconversion | 30 | 24 | 0.000 | 0.047 | 4 | 0.000 | 0.219 | 3 |
|  | lmo0096 | 16802144 | Multiple Roles | Multiple Roles | 7 | 5 | 0.000 | 0.160 | 2 | 0.000 | 0.010 | 2 |
| hypothetical protein lmo0098 | lmo0098 | 16802146 | Multiple Roles | Multiple Roles | 12 | 5 | 0.069 | 0.152 | 2 | 0.092 | 0.020 | 2 |
|  | lmo0100 | 16802148 | Function Unknown | Unknown General | 5 | 8 | 0.000 | 0.100 | 2 | 0.000 | 0.000 | 2 |
|  | lmo0114 | 16802162 | Mobile and Extrachromosomal Element Functions | Prophage Functions | 9 | 10 | 0.069 | 0.099 | 2 | 0.092 | 0.222 | 2 |
| hypothetical protein lmo0130 | lmo0130 | 16802178 | Cell Envelope | Other | 22 | 28 | 0.076 | 0.115 | 3 | 0.101 | 0.000 | 4 |
| hypothetical protein lmo0135 | lmo0135 | 16802183 | Transport and Binding Proteins | Amino Acids, Peptides and Amines | 35 | 29 | 0.076 | 0.001 | 5 | 0.066 | 0.064 | 4 |
|  | lmo0152 | 16802200 | Multiple Roles | Multiple Roles | 7 | 7 | 0.076 | 0.015 | 2 | 0.101 | 0.096 | 2 |
|  | lmo0163 | 16802211 | Function Unknown | Unknown General | 5 | 6 | 0.000 | 0.000 | 2 | 0.000 | 0.144 | 2 |
|  | lmo0168 | 16802216 | Regulatory Functions | Other | 5 | 7 | 0.064 | 0.299 | 2 | 0.069 | 0.056 | 2 |
| methionyl-tRNA synthetase | lmo0177 | 16802223 | Protein Synthesis | tRNA Aminoacylation | 19 | 11 | 0.008 | 0.048 | 3 | 0.101 | 0.000 | 2 |
| hypothetical protein lmo0181 | lmo0181 | 16802227 | Transport and Binding Proteins | Unknown Substrate | 11 | 36 | 0.000 | 0.030 | 2 | 0.000 | 0.000 | 5 |
|  | lmo0182 | 16802228 | Energy Metabolism | Sugars | 6 | 6 | 0.000 | 0.100 | 2 | 0.000 | 0.006 | 2 |
|  | lmo0183 | 16802229 | Energy Metabolism | Sugars | 6 | 9 | 0.069 | 0.000 | 2 | 0.092 | 0.258 | 2 |
| hypothetical protein lmo0184 | lmo0184 | 16802230 | Energy Metabolism | Sugars | 11 | 38 | 0.000 | 0.085 | 2 | 0.000 | 0.000 | 5 |
|  | lmo0191 | 16802237 | Multiple Roles | Multiple Roles | 13 | 11 | 0.000 | 0.001 | 2 | 0.000 | 0.001 | 2 |
| regulatory protein SpoVG | lmo0197 | 16802243 | Cellular Processes | Adaptations to Atypical Conditions | 115 | 206 | 0.000 | 0.246 | 13 | 0.092 | 0.093 | 22 |
|  | lmo0198 | 16802244 | Cell Envelope | Biosynthesis and Degradation of Surface Polysaccharides and Lipopolysaccharides | 5 | 8 | 0.000 | 0.006 | 2 | 0.000 | 0.127 | 2 |
|  | lmo0199 | 16802245 | Purines, Pyrimidines, Nucleosides and Nucleotides | Purine Ribonucleotide Biosynthesis | 13 | 13 | 0.000 | 0.042 | 2 | 0.000 | 0.221 | 2 |
|  | lmo0201 | 16802247 | Multiple Roles | Multiple Roles | 5 | 8 | 0.000 | 0.000 | 2 | 0.000 | 0.022 | 2 |
| listeriolysin O precursor | lmo0202 | 16802248 | Cellular Processes | Pathogenesis | 53 | 45 | 0.000 | 0.000 | 6 | 0.000 | 0.000 | 6 |
|  | lmo0203 | 16802249 | Protein Fate | Degradation of Proteins, Peptides and Glycopeptides | 5 | 6 | 0.010 | 0.089 | 2 | 0.000 | 0.000 | 2 |
| actin-assembly inducing protein precursor (actA) | lmo0204 | 16802250 | Cellular Processes | Pathogenesis | 5 | 16 | 0.000 | 0.018 | 2 | 0.000 | 0.234 | 3 |
|  | lmo0210 | 16802256 | Multiple Roles | Multiple Roles | 33 | 39 | 0.000 | 0.000 | 4 | 0.000 | 0.009 | 5 |
| 50S ribosomal protein L25/general stress protein Ctc | lmo0211 | 16802257 | Protein Synthesis | Ribosomal Proteins: Synthesis and Modification | 26 | 20 | 0.000 | 0.043 | 3 | 0.092 | 0.222 | 4 |
|  | lmo0216 | 16802262 | Protein Fate | Protein Folding and Stabilisation | 5 | 7 | 0.000 | 0.000 | 2 | 0.000 | 0.000 | 2 |
| hypothetical protein lmo0218 | lmo0218 | 16802264 | Transcription | Degradation of RNA | 10 | 25 | 0.000 | 0.102 | 2 | 0.000 | 0.085 | 4 |
| bifunctional protein TilS/HprT | lmo0219 | 16802265 | Purines, Pyrimidines, Nucleosides and Nucleotides | Salvage of Nucleotides and Nucleosides | 5 | 12 | 0.000 | 0.123 | 2 | 0.000 | 0.246 | 2 |
| cell division protease FtsH | lmo0220 | 16802266 | Cellular Processes | Cell Division | 5 | 10 | 0.000 | 0.001 | 2 | 0.000 | 0.000 | 2 |
| hypothetical protein lmo0223 | lmo0223 | 16802269 | Amino Acid Biosynthesis | Serine Family | 54 | 32 | 0.069 | 0.291 | 6 | 0.092 | 0.077 | 4 |
|  | lmo0225 | 16802271 | Biosynthesis of Cofactors, Prosthetic Groups and Carriers | Folate | 8 | 5 | 0.003 | 0.118 | 2 | 0.004 | 0.247 | 2 |
| lysyl-tRNA synthetase - | lmo0228 | 16802274 | Protein Synthesis | tRNA Aminoacylation | 13 | 7 | 0.000 | 0.299 | 2 | 0.092 | 0.196 | 2 |
| endopeptidase Clp ATP-binding chain C | lmo0232 | 16802278 | Multiple Roles | Multiple Roles | 16 | 5 | 0.000 | 0.125 | 3 | 0.000 | 0.004 | 2 |
|  | lmo0236 | 16802282 | Biosynthesis of Cofactors, Prosthetic Groups and Carriers | Other | 8 | 5 | 0.000 | 0.102 | 2 | 0.000 | 0.213 | 2 |
| hypothetical protein lmo0237 - | lmo0237 | 16802283 | Protein Synthesis | tRNA Aminoacylation | 11 | 6 | 0.000 | 0.146 | 2 | 0.000 | 0.051 | 2 |
|  | lmo0239 | 16802285 | Protein Synthesis | tRNA Aminoacylation | 5 | 6 | 0.000 | 0.207 | 2 | 0.000 | 0.010 | 2 |
| transcription antitermination protein NusG | lmo0246 | 16802292 | Transcription | Transcription Factors | 11 | 6 | 0.076 | 0.071 | 2 | 0.000 | 0.000 | 2 |
|  | lmo0248 | 16802294 | Protein Synthesis | Ribosomal Proteins: Synthesis and Modification | 9 | 11 | 0.000 | 0.252 | 2 | 0.092 | 0.020 | 2 |
| 50S ribosomal protein L1 | lmo0249 | 16802295 | Protein Synthesis | Ribosomal Proteins: Synthesis and Modification | 20 | 5 | 0.076 | 0.219 | 2 | 0.101 | 0.096 | 3 |
| 50S ribosomal protein L10 | lmo0250 | 16802296 | Protein Synthesis | Ribosomal Proteins: Synthesis and Modification | 32 | 10 | 0.000 | 0.030 | 2 | 0.000 | 0.001 | 4 |
| large subunit ribosomal protein L7/L12 | lmo0251 | 16802297 | Protein Synthesis | Ribosomal Proteins: Synthesis and Modification | 60 | 37 | 0.000 | 0.185 | 5 | 0.000 | 0.022 | 7 |
|  | lmo0258 | 16802304 | Transcription | DNA Dependant RNA Polymerase | 18 | 21 | 0.000 | 0.000 | 3 | 0.000 | 0.233 | 3 |
|  | lmo0261 | 16802307 | Energy Metabolism | Sugars | 6 | 5 | 0.000 | 0.000 | 2 | 0.000 | 0.000 | 2 |
| probable phosphoglycerate mutase | lmo0268 | 16802314 | Energy Metabolism | Glycolysis/Gluconeogenesis | 54 | 75 | 0.000 | 0.001 | 6 | 0.000 | 0.001 | 9 |
|  | lmo0271 | 16802317 | Central Intermediary Metabolism | Other | 9 | 5 | 0.000 | 0.070 | 2 | 0.000 | 0.061 | 2 |
|  | lmo0273 | 16802319 | Function Unknown | Enzyme of Unknown Specificity | 5 | 6 | 0.001 | 0.003 | 2 | 0.001 | 0.001 | 2 |
|  | lmo0278 | 16802324 | Transport and Binding Proteins | Carbohydrates, Organic Alcohols and Acids | 13 | 12 | 0.000 | 0.000 | 2 | 0.000 | 0.080 | 2 |
| hypothetical protein lmo0287 | lmo0287 | 16802333 | Multiple Roles | Multiple Roles | 12 | 5 | 0.000 | 0.001 | 2 | 0.000 | 0.002 | 2 |
|  | lmo0288 | 16802334 | Regulatory Functions | Other | 6 | 5 | 0.064 | 0.292 | 2 | 0.000 | 0.003 | 2 |
| hypothetical protein lmo0292 | lmo0292 | 16802338 | Protein Fate | Degradation of Proteins, Peptides and Glycopeptides | 9 | 5 | 0.000 | 0.102 | 2 | 0.092 | 0.255 | 2 |
|  | lmo0302 | 16802348 | Function Unknown | Unknown General | 6 | 7 | 0.000 | 0.000 | 2 | 0.000 | 0.036 | 2 |
| hypothetical protein lmo0319 | lmo0319 | 16802364 | Energy Metabolism | Sugars | 24 | 46 | 0.000 | 0.233 | 3 | 0.000 | 0.000 | 6 |
|  | lmo0322 | 16802367 | Function Unknown | Unknown General | 5 | 6 | 0.000 | 0.000 | 2 | 0.000 | 0.008 | 2 |
|  | lmo0333 | 16802378 | Cellular Processes | Pathogenesis | 5 | 7 | 0.000 | 0.088 | 2 | 0.000 | 0.029 | 2 |
|  | lmo0348 | 16802393 | Multiple Roles | Multiple Roles | 6 | 5 | 0.000 | 0.076 | 2 | 0.000 | 0.000 | 2 |
| fumarate reductase flavoprotein subunit | lmo0355 | 16802400 | Function Unknown | Unknown General | 63 | 35 | 0.000 | 0.001 | 5 | 0.000 | 0.212 | 7 |
|  | lmo0356 | 16802401 | Multiple Roles | Multiple Roles | 5 | 6 | 0.000 | 0.042 | 2 | 0.000 | 0.000 | 2 |
|  | lmo0370 | 16802415 | Central Intermediary Metabolism | Phosphorous Compounds | 9 | 10 | 0.069 | 0.006 | 2 | 0.092 | 0.042 | 2 |
|  | lmo0387 | 16802432 | Protein Fate | Degradation of Proteins, Peptides and Glycopeptides | 10 | 10 | 0.076 | 0.071 | 2 | 0.011 | 0.116 | 2 |
|  | lmo0391 | 16802436 | Function Unknown | Unknown General | 12 | 11 | 0.000 | 0.001 | 2 | 0.000 | 0.086 | 2 |
| Hypothetical protein | lmo0392 | 16802437 | Function Unknown | Unknown General | 5 | 12 | 0.001 | 0.134 | 2 | 0.001 | 0.000 | 2 |
|  | lmo0394 | 16802439 | Cellular Processes | Pathogenesis | 6 | 5 | 0.000 | 0.003 | 2 | 0.000 | 0.002 | 2 |
| hypothetical protein lmo0427 | lmo0427 | 16802471 | Regulatory Functions | Other | 8 | 24 | 0.000 | 0.285 | 2 | 0.076 | 0.034 | 3 |
|  | lmo0429 | 16802473 | Function Unknown | Unknown General | 7 | 5 | 0.000 | 0.005 | 2 | 0.000 | 0.208 | 2 |
|  | lmo0433 | 16802477 | Cellular Processes | Pathogenesis | 7 | 5 | 0.000 | 0.000 | 2 | 0.000 | 0.000 | 2 |
|  | lmo0437 | 16802481 | Function Unknown | Unknown General | 6 | 5 | 0.076 | 0.130 | 2 | 0.101 | 0.250 | 2 |
|  | lmo0441 | 16802485 | Cell Envelope | Biosynthesis and Degradation of Murein Sacculus and Peptidoglycan | 5 | 6 | 0.001 | 0.140 | 2 | 0.092 | 0.158 | 2 |
|  | lmo0443 | 16802487 | Regulatory Functions | Other | 5 | 7 | 0.007 | 0.284 | 2 | 0.000 | 0.255 | 2 |
|  | lmo0447 | 16802491 | Multiple Roles | Multiple Roles | 8 | 5 | 0.000 | 0.123 | 2 | 0.000 | 0.029 | 2 |
|  | lmo0454 | 16802498 | Multiple Roles | Multiple Roles | 5 | 6 | 0.000 | 0.053 | 2 | 0.000 | 0.000 | 2 |
|  | lmo0479 | 16802522 | Function Unknown | Unknown General | 10 | 6 | 0.076 | 0.298 | 2 | 0.101 | 0.024 | 2 |
|  | lmo0484 | 16802527 | Function Unknown | Unknown General | 5 | 6 | 0.001 | 0.154 | 2 | 0.001 | 0.000 | 2 |
|  | lmo0491 | 16802534 | Amino Acid Biosynthesis | Aromatic Amino Acid | 5 | 6 | 0.000 | 0.279 | 2 | 0.000 | 0.010 | 2 |
| PTS system, galactitol-specific IIB component | lmo0507 | 16802550 | Transport and Binding Proteins | Carbohydrates, Organic Alcohols and Acids | 5 | 10 | 0.000 | 0.125 | 2 | 0.004 | 0.003 | 2 |
| hypothetical protein lmo0517 | lmo0517 | 16802560 | Energy Metabolism | Glycolysis/Gluconeogenesis | 7 | 44 | 0.000 | 0.001 | 2 | 0.000 | 0.000 | 5 |
|  | lmo0530 | 16802573 | Function Unknown | Unknown General | 6 | 5 | 0.000 | 0.276 | 2 | 0.000 | 0.000 | 2 |
|  | lmo0535 | 16802578 | Regulatory Functions | Other | 6 | 5 | 0.003 | 0.191 | 2 | 0.000 | 0.000 | 2 |
| 6-phospho-beta-glucosidase | lmo0536 | 16802579 | Central Intermediary Metabolism | Other | 10 | 14 | 0.069 | 0.052 | 2 | 0.092 | 0.254 | 2 |
|  | lmo0553 | 16802596 | Function Unknown | Unknown General | 6 | 7 | 0.004 | 0.052 | 2 | 0.005 | 0.000 | 2 |
|  | lmo0558 | 16802601 | Function Unknown | Unknown General | 5 | 7 | 0.000 | 0.084 | 2 | 0.015 | 0.002 | 2 |
|  | lmo0559 | 16802602 | Transport and Binding Proteins | Unknown Substrate | 6 | 5 | 0.000 | 0.252 | 2 | 0.000 | 0.006 | 2 |
|  | lmo0579 | 16802622 | Function Unknown | Unknown General | 8 | 7 | 0.000 | 0.022 | 2 | 0.000 | 0.003 | 2 |
|  | lmo0600 | 16802642 | Function Unknown | Unknown General | 6 | 5 | 0.069 | 0.240 | 2 | 0.092 | 0.018 | 2 |
| hypothetical protein lmo0640 | lmo0640 | 16802682 | Multiple Roles | Multiple Roles | 20 | 5 | 0.008 | 0.048 | 3 | 0.011 | 0.063 | 2 |
|  | lmo0662 | 16802704 | Biosynthesis of Cofactors, Prosthetic Groups and Carriers | Thiamine | 8 | 6 | 0.064 | 0.226 | 2 | 0.101 | 0.089 | 2 |
|  | lmo0663 | 16802705 | Central Intermediary Metabolism | Other | 6 | 5 | 0.000 | 0.020 | 2 | 0.000 | 0.044 | 2 |
|  | lmo0673 | 16802715 | Function Unknown | Unknown General | 6 | 8 | 0.000 | 0.047 | 2 | 0.000 | 0.000 | 2 |
| flagellin | lmo0690 | 16802732 | Cellular Processes | Chemotaxis and Motility | 20 | 5 | 0.000 | 0.084 | 3 | 0.000 | 0.000 | 2 |
|  | lmo0702 | 16802744 | Function Unknown | Unknown General | 5 | 6 | 0.000 | 0.000 | 2 | 0.000 | 0.000 | 2 |
|  | lmo0713 | 16802755 | Cellular Processes | Chemotaxis and Motility | 5 | 6 | 0.000 | 0.012 | 2 | 0.000 | 0.000 | 2 |
|  | lmo0719 | 16802761 | Function Unknown | Unknown General | 5 | 6 | 0.000 | 0.038 | 2 | 0.000 | 0.000 | 2 |
| glucosamine--fructose-6-phosphate aminotransferase | lmo0727 | 16802769 | Central Intermediary Metabolism | Amino Sugars | 17 | 24 | 0.000 | 0.257 | 3 | 0.013 | 0.000 | 3 |
|  | lmo0774 | 16802816 | Function Unknown | Unknown General | 7 | 6 | 0.000 | 0.195 | 2 | 0.000 | 0.174 | 2 |
|  | lmo0775 | 16802817 | Function Unknown | Unknown General | 9 | 13 | 0.069 | 0.030 | 2 | 0.092 | 0.011 | 2 |
|  | lmo0785 | 16802827 | Regulatory Functions | Other | 8 | 6 | 0.064 | 0.184 | 2 | 0.000 | 0.013 | 2 |
| hypothetical protein lmo0786 | lmo0786 | 16802828 | Fatty Acid and Phospholipid Metabolism | Other | 22 | 13 | 0.000 | 0.028 | 3 | 0.092 | 0.035 | 2 |
|  | lmo0791 | 16802833 | Function Unknown | Unknown General | 7 | 5 | 0.000 | 0.146 | 2 | 0.101 | 0.151 | 2 |
|  | lmo0796 | 16802838 | Function Unknown | Unknown General | 7 | 5 | 0.000 | 0.161 | 2 | 0.092 | 0.158 | 2 |
|  | lmo0797 | 16802839 | Regulatory Functions | DNA Interactions | 5 | 7 | 0.000 | 0.203 | 2 | 0.000 | 0.001 | 2 |
|  | lmo0810 | 16802852 | Transport and Binding Proteins | Amino Acids, Peptides and Amines | 6 | 5 | 0.000 | 0.001 | 2 | 0.000 | 0.021 | 2 |
|  | lmo0814 | 16802856 | Function Unknown | Unknown General | 5 | 6 | 0.076 | 0.002 | 2 | 0.101 | 0.225 | 2 |
|  | lmo0830 | 16802872 | Energy Metabolism | Glycolysis/Gluconeogenesis | 5 | 6 | 0.000 | 0.233 | 2 | 0.000 | 0.000 | 2 |
|  | lmo0842 | 16802883 | Protein Fate | Protein and Peptide secretion and Trafficking | 6 | 5 | 0.000 | 0.000 | 2 | 0.092 | 0.248 | 2 |
|  | lmo0844 | 16802885 | Transcription | Degradation of RNA | 5 | 7 | 0.069 | 0.003 | 2 | 0.000 | 0.139 | 2 |
|  | lmo0859 | 16802900 | Transport and Binding Proteins | Carbohydrates, Organic Alcohols and Acids | 5 | 6 | 0.000 | 0.001 | 2 | 0.000 | 0.021 | 2 |
| hypothetical protein lmo0866 | lmo0866 | 16802907 | Multiple Roles | Multiple Roles | 14 | 5 | 0.000 | 0.043 | 2 | 0.000 | 0.000 | 2 |
| hypothetical protein lmo0880 | lmo0880 | 16802921 | Cell Envelope | Other | 5 | 16 | 0.069 | 0.001 | 2 | 0.092 | 0.063 | 2 |
|  | lmo0885 | 16802926 | Fatty Acid and Phospholipid Metabolism | Biosynthesis | 5 | 9 | 0.000 | 0.003 | 2 | 0.000 | 0.000 | 2 |
| mRNA interferase | lmo0888 | 16802929 | Regulatory Functions | Other | 5 | 13 | 0.005 | 0.031 | 2 | 0.069 | 0.107 | 2 |
|  | lmo0891 | 16802932 | Transcription | Transcription Factors | 5 | 6 | 0.000 | 0.003 | 2 | 0.004 | 0.019 | 2 |
|  | lmo0894 | 16802935 | Transcription | Transcription Factors | 10 | 6 | 0.000 | 0.000 | 2 | 0.000 | 0.040 | 2 |
|  | lmo0898 | 16802939 | Multiple Roles | Multiple Roles | 7 | 5 | 0.000 | 0.299 | 2 | 0.000 | 0.000 | 2 |
|  | lmo0900 | 16802941 | Function Unknown | Unknown General | 5 | 8 | 0.000 | 0.251 | 2 | 0.000 | 0.026 | 2 |
|  | lmo0903 | 16802944 | Function Unknown | Unknown General | 9 | 6 | 0.000 | 0.002 | 2 | 0.001 | 0.201 | 2 |
|  | lmo0907 | 16802948 | Energy Metabolism | Glycolysis/Gluconeogenesis | 9 | 7 | 0.066 | 0.064 | 2 | 0.092 | 0.161 | 2 |
|  | lmo0925 | 16802965 | Transport and Binding Proteins | Unknown Substrate | 6 | 5 | 0.000 | 0.207 | 2 | 0.069 | 0.065 | 2 |
| hypothetical protein lmo0930 | lmo0930 | 16802970 | Function Unknown | Unknown General | 12 | 5 | 0.000 | 0.000 | 2 | 0.000 | 0.000 | 2 |
| non-heme iron-binding ferritin | lmo0943 | 16802983 | Energy Metabolism | Fermentation | 8 | 18 | 0.000 | 0.021 | 2 | 0.000 | 0.007 | 2 |
|  | lmo0955 | 16802995 | Function Unknown | Unknown General | 6 | 10 | 0.076 | 0.293 | 2 | 0.101 | 0.183 | 2 |
|  | lmo0956 | 16802996 | Central Intermediary Metabolism | Amino Sugars | 7 | 5 | 0.069 | 0.056 | 2 | 0.092 | 0.013 | 2 |
|  | lmo0961 | 16803001 | Protein Fate | Degradation of Proteins, Peptides and Glycopeptides | 5 | 6 | 0.000 | 0.016 | 2 | 0.000 | 0.001 | 2 |
| epitope LemA | lmo0962 | 16803002 | Function Unknown | Unknown General | 8 | 20 | 0.000 | 0.010 | 2 | 0.000 | 0.000 | 3 |
|  | lmo0963 | 16803003 | Protein Fate | Protein Folding and Stabilisation | 7 | 6 | 0.000 | 0.023 | 2 | 0.000 | 0.009 | 2 |
|  | lmo0968 | 16803008 | Biosynthesis of Cofactors, Prosthetic Groups and Carriers | Pyridine Nucleotides | 9 | 7 | 0.000 | 0.262 | 2 | 0.000 | 0.060 | 2 |
| enoyl-(acyl carrier protein) reductase | lmo0970 | 16803010 | Fatty Acid and Phospholipid Metabolism | Biosynthesis | 13 | 7 | 0.000 | 0.169 | 2 | 0.000 | 0.001 | 2 |
| D-alanine--poly(phosphoribitol) ligase subunit 2 | lmo0972 | 16803012 | Cell Envelope | Biosynthesis and Degradation of Murein Sacculus and Peptidoglycan | 21 | 5 | 0.066 | 0.000 | 3 | 0.092 | 0.253 | 2 |
| D-alanine--D-alanyl carrier protein ligase | lmo0974 | 16803014 | Function Unknown | Unknown General | 7 | 13 | 0.000 | 0.000 | 2 | 0.000 | 0.000 | 2 |
|  | lmo0978 | 16803018 | Amino Acid Biosynthesis | Pyruvate Family | 12 | 18 | 0.076 | 0.192 | 2 | 0.101 | 0.257 | 3 |
| hypothetical protein lmo1001 | lmo1001 | 16803041 | Function Unknown | Unknown General | 9 | 22 | 0.000 | 0.009 | 2 | 0.000 | 0.001 | 3 |
| phosphocarrier protein HPr | lmo1002 | 16803042 | Transport and Binding Proteins | Carbohydrates, Organic Alcohols and Acids | 236 | 350 | 0.000 | 0.076 | 25 | 0.001 | 0.000 | 16 |
| phosphotransferase system, enzyme I, PtsI | lmo1003 | 16803043 | Transport and Binding Proteins | Carbohydrates, Organic Alcohols and Acids | 19 | 34 | 0.000 | 0.042 | 3 | 0.069 | 0.033 | 4 |
|  | lmo1005 | 16803045 | Multiple Roles | Multiple Roles | 6 | 5 | 0.000 | 0.146 | 2 | 0.000 | 0.022 | 2 |
|  | lmo1011 | 16803051 | Amino Acid Biosynthesis | Aspartate-Family | 9 | 5 | 0.000 | 0.010 | 2 | 0.000 | 0.002 | 2 |
|  | lmo1014 | 16803054 | Transport and Binding Proteins | Amino Acids, Peptides and Amines | 8 | 6 | 0.076 | 0.015 | 2 | 0.064 | 0.001 | 2 |
|  | lmo1017 | 16803057 | Transport and Binding Proteins | Carbohydrates, Organic Alcohols and Acids | 5 | 6 | 0.000 | 0.123 | 2 | 0.000 | 0.169 | 2 |
|  | lmo1027 | 16803067 | Multiple Roles | Multiple Roles | 5 | 7 | 0.000 | 0.207 | 2 | 0.000 | 0.035 | 2 |
| hypothetical protein lmo1028 | lmo1028 | 16803068 | Function Unknown | Unknown General | 18 | 71 | 0.000 | 0.000 | 3 | 0.000 | 0.000 | 8 |
|  | lmo1051 | 16803091 | Protein Fate | Protein Modification and Repair | 7 | 6 | 0.000 | 0.187 | 2 | 0.000 | 0.016 | 2 |
| pyruvate dehydrogenase E1 component subunit alpha | lmo1052 | 16803092 | Energy Metabolism | Pyruvate Dehydrogenase | 26 | 38 | 0.000 | 0.039 | 4 | 0.000 | 0.251 | 5 |
| pyruvate dehydrogenase E1 component subunit beta | lmo1053 | 16803093 | Energy Metabolism | Pyruvate Dehydrogenase | 16 | 25 | 0.000 | 0.080 | 3 | 0.000 | 0.000 | 4 |
|  | lmo1059 | 16803099 | Function Unknown | Unknown General | 13 | 17 | 0.000 | 0.000 | 2 | 0.000 | 0.000 | 3 |
|  | lmo1060 | 16803100 | Regulatory Functions | Other | 5 | 6 | 0.000 | 0.078 | 2 | 0.000 | 0.000 | 2 |
|  | lmo1067 | 16803107 | Function Unknown | Unknown General | 5 | 7 | 0.000 | 0.037 | 2 | 0.000 | 0.036 | 2 |
| pyruvate carboxylase | lmo1072 | 16803112 | Energy Metabolism | Glycolysis/Gluconeogenesis | 9 | 16 | 0.000 | 0.183 | 2 | 0.000 | 0.000 | 3 |
|  | lmo1075 | 16803115 | Function Unknown | Unknown General | 5 | 6 | 0.000 | 0.000 | 2 | 0.000 | 0.000 | 2 |
|  | lmo1078 | 16803118 | Cell Envelope | Biosynthesis and Degradation of Surface Polysaccharides and Lipopolysaccharides | 11 | 8 | 0.000 | 0.094 | 2 | 0.000 | 0.000 | 2 |
|  | lmo1093 | 16803133 | Biosynthesis of Cofactors, Prosthetic Groups and Carriers | Pyridine Nucleotides | 6 | 8 | 0.000 | 0.158 | 2 | 0.000 | 0.008 | 2 |
|  | lmo1096 | 16803136 | Purines, Pyrimidines, Nucleosides and Nucleotides | Purine Ribonucleotide Biosynthesis | 11 | 14 | 0.000 | 0.053 | 2 | 0.076 | 0.295 | 2 |
|  | lmo1159 | 16803199 | Function Unknown | Unknown General | 5 | 6 | 0.000 | 0.004 | 2 | 0.000 | 0.197 | 2 |
|  | lmo1168 | 16803208 | Multiple Roles | Multiple Roles | 16 | 14 | 0.000 | 0.252 | 3 | 0.000 | 0.007 | 2 |
|  | lmo1180 | 16803220 | Function Unknown | Unknown General | 5 | 8 | 0.000 | 0.000 | 2 | 0.000 | 0.000 | 2 |
|  | lmo1213 | 16803253 | Function Unknown | Unknown General | 6 | 6 | 0.000 | 0.000 | 2 | 0.000 | 0.000 | 2 |
| hypothetical protein lmo1217 | lmo1217 | 16803257 | Protein Fate | Degradation of Proteins, Peptides and Glycopeptides | 17 | 7 | 0.076 | 0.258 | 3 | 0.000 | 0.026 | 2 |
|  | lmo1222 | 16803262 | Protein Synthesis | tRNA Aminoacylation | 6 | 5 | 0.004 | 0.271 | 2 | 0.000 | 0.009 | 2 |
|  | lmo1226 | 16803266 | Cell Envelope | Other | 6 | 5 | 0.000 | 0.253 | 2 | 0.000 | 0.239 | 2 |
| hypothetical protein lmo1229 | lmo1229 | 16803269 | Function Unknown | Unknown General | 23 | 54 | 0.000 | 0.000 | 3 | 0.000 | 0.000 | 6 |
| thioredoxin | lmo1233 | 16803273 | Energy Metabolism | Electron Transport | 20 | 6 | 0.001 | 0.000 | 2 | 0.001 | 0.255 | 2 |
|  | lmo1236 | 16803276 | Function Unknown | Unknown General | 6 | 5 | 0.000 | 0.003 | 2 | 0.000 | 0.000 | 2 |
|  | lmo1240 | 16803280 | Function Unknown | Enzyme of Unknown Specificity | 9 | 6 | 0.000 | 0.207 | 2 | 0.000 | 0.015 | 2 |
| hypothetical protein lmo1249 | lmo1249 | 16803289 | Function Unknown | Unknown General | 12 | 5 | 0.000 | 0.029 | 2 | 0.000 | 0.014 | 2 |
|  | lmo1254 | 16803294 | Energy Metabolism | TCA Cycle | 6 | 6 | 0.000 | 0.169 | 2 | 0.000 | 0.146 | 2 |
|  | lmo1257 | 16803297 | Function Unknown | Unknown General | 7 | 7 | 0.000 | 0.000 | 2 | 0.000 | 0.097 | 2 |
|  | lmo1262 | 16803302 | Regulatory Functions | Other | 5 | 6 | 0.008 | 0.236 | 2 | 0.064 | 0.043 | 2 |
| trigger factor | lmo1267 | 16803307 | Multiple Roles | Multiple Roles | 30 | 16 | 0.076 | 0.219 | 3 | 0.101 | 0.050 | 4 |
|  | lmo1268 | 16803308 | Protein Fate | Degradation of Proteins, Peptides and Glycopeptides | 7 | 8 | 0.000 | 0.000 | 2 | 0.000 | 0.039 | 2 |
|  | lmo1279 | 16803319 | Protein Fate | Protein Folding and Stabilisation | 7 | 5 | 0.000 | 0.224 | 2 | 0.000 | 0.000 | 2 |
| transcriptional repressor CodY | lmo1280 | 16803320 | Regulatory Functions | Other | 5 | 15 | 0.064 | 0.162 | 2 | 0.000 | 0.140 | 3 |
|  | lmo1283 | 16803323 | Function Unknown | Unknown General | 8 | 6 | 0.069 | 0.162 | 2 | 0.092 | 0.000 | 2 |
|  | lmo1285 | 16803325 | Multiple Roles | Multiple Roles | 5 | 7 | 0.000 | 0.030 | 2 | 0.011 | 0.258 | 2 |
|  | lmo1286 | 16803326 | DNA Metabolism | DNA Replication, Recombination and Repair | 5 | 6 | 0.009 | 0.293 | 2 | 0.101 | 0.250 | 2 |
|  | lmo1291 | 16803331 | Function Unknown | Unknown General | 5 | 6 | 0.004 | 0.000 | 2 | 0.006 | 0.003 | 2 |
| phosphodiesterase | lmo1292 | 16803332 | Multiple Roles | Multiple Roles | 9 | 16 | 0.000 | 0.185 | 2 | 0.101 | 0.172 | 3 |
|  | lmo1293 | 16803333 | Energy Metabolism | Other | 9 | 8 | 0.000 | 0.017 | 2 | 0.000 | 0.000 | 2 |
|  | lmo1295 | 16803335 | Multiple Roles | Multiple Roles | 5 | 7 | 0.004 | 0.271 | 2 | 0.007 | 0.000 | 2 |
| hypothetical protein lmo1298 | lmo1298 | 16803338 | Regulatory Functions | Other | 9 | 19 | 0.064 | 0.103 | 2 | 0.064 | 0.144 | 3 |
|  | lmo1299 | 16803339 | Amino Acid Biosynthesis | Glutatmate-Family | 13 | 9 | 0.000 | 0.284 | 2 | 0.092 | 0.228 | 2 |
|  | lmo1302 | 16803342 | Multiple Roles | Multiple Roles | 5 | 6 | 0.000 | 0.001 | 2 | 0.101 | 0.017 | 2 |
| hypothetical protein lmo1305 | lmo1305 | 16803345 | Energy Metabolism | Pentose Phosphate pathway | 13 | 28 | 0.000 | 0.010 | 2 | 0.000 | 0.000 | 4 |
| ribosome recycling factor | lmo1314 | 16803354 | Protein Synthesis | Other | 5 | 16 | 0.000 | 0.001 | 2 | 0.000 | 0.004 | 3 |
|  | lmo1317 | 16803357 | Biosynthesis of Cofactors, Prosthetic Groups and Carriers | Other | 7 | 5 | 0.000 | 0.191 | 2 | 0.000 | 0.000 | 2 |
|  | lmo1319 | 16803359 | Protein Synthesis | tRNA Aminoacylation | 9 | 12 | 0.000 | 0.153 | 2 | 0.076 | 0.212 | 2 |
|  | lmo1321 | 16803361 | Function Unknown | Unknown General | 6 | 5 | 0.000 | 0.281 | 2 | 0.101 | 0.000 | 2 |
| transcription elongation factor NusA | lmo1322 | 16803362 | Transcription | Transcription Factors | 10 | 5 | 0.000 | 0.023 | 2 | 0.000 | 0.000 | 2 |
|  | lmo1323 | 16803363 | Function Unknown | Unknown General | 6 | 6 | 0.000 | 0.173 | 2 | 0.092 | 0.245 | 2 |
|  | lmo1324 | 16803364 | Function Unknown | Unknown General | 5 | 7 | 0.000 | 0.238 | 2 | 0.101 | 0.197 | 2 |
|  | lmo1325 | 16803365 | Protein Synthesis | Other | 6 | 5 | 0.000 | 0.146 | 2 | 0.076 | 0.002 | 2 |
| ribosome-binding factor A | lmo1327 | 16803367 | Transcription | RNA Processing | 6 | 25 | 0.000 | 0.224 | 2 | 0.000 | 0.000 | 4 |
| 30S ribosomal protein S15 | lmo1330 | 16803370 | Protein Synthesis | Ribosomal Proteins: Synthesis and Modification | 32 | 21 | 0.000 | 0.054 | 3 | 0.064 | 0.000 | 4 |
| polynucleotide phosphorylase/polyadenylase | lmo1331 | 16803371 | Transcription | Degradation of RNA | 11 | 6 | 0.000 | 0.187 | 2 | 0.000 | 0.001 | 2 |
|  | lmo1336 | 16803376 | Function Unknown | Unknown General | 6 | 6 | 0.000 | 0.000 | 2 | 0.092 | 0.132 | 2 |
|  | lmo1338 | 16803378 | Function Unknown | Unknown General | 6 | 8 | 0.000 | 0.123 | 2 | 0.000 | 0.095 | 2 |
| glycine dehydrogenase subunit 1 | lmo1349 | 16803389 | Multiple Roles | Multiple Roles | 5 | 9 | 0.000 | 0.153 | 2 | 0.101 | 0.242 | 2 |
|  | lmo1351 | 16803391 | Central Intermediary Metabolism | Sulphur Metabolism | 7 | 5 | 0.000 | 0.003 | 2 | 0.000 | 0.011 | 2 |
| hypothetical protein lmo1354 | lmo1354 | 16803394 | Protein Fate | Degradation of Proteins, Peptides and Glycopeptides | 13 | 6 | 0.000 | 0.000 | 2 | 0.000 | 0.000 | 2 |
| elongation factor P | lmo1355 | 16803395 | Protein Synthesis | Other | 11 | 5 | 0.064 | 0.299 | 2 | 0.000 | 0.010 | 2 |
|  | lmo1357 | 16803397 | Fatty Acid and Phospholipid Metabolism | Biosynthesis | 5 | 9 | 0.000 | 0.294 | 2 | 0.000 | 0.001 | 2 |
| hypothetical protein lmo1360 | lmo1360 | 16803400 | Biosynthesis of Cofactors, Prosthetic Groups and Carriers | Folate | 10 | 5 | 0.005 | 0.001 | 2 | 0.007 | 0.058 | 2 |
|  | lmo1362 | 16803402 | DNA Metabolism | Degradation of DNA | 5 | 6 | 0.069 | 0.003 | 2 | 0.092 | 0.148 | 2 |
| hypothetical protein lmo1364 | lmo1364 | 16803404 | Cellular Processes | Adaptations to Atypical Conditions | 198 | 89 | 0.000 | 0.036 | 11 | 0.000 | 0.129 | 10 |
|  | lmo1367 | 16803407 | Regulatory Functions | Other | 7 | 5 | 0.000 | 0.000 | 2 | 0.009 | 0.293 | 2 |
| dihydrolipoamide dehydrogenase | lmo1371 | 16803411 | Energy Metabolism | Pyruvate Dehydrogenase | 25 | 14 | 0.004 | 0.003 | 4 | 0.005 | 0.104 | 2 |
|  | lmo1372 | 16803412 | Transport and Binding Proteins | Amino Acids, Peptides and Amines | 5 | 6 | 0.069 | 0.152 | 2 | 0.000 | 0.079 | 2 |
|  | lmo1373 | 16803413 | Energy Metabolism | Pyruvate Dehydrogenase | 8 | 9 | 0.000 | 0.021 | 2 | 0.000 | 0.000 | 2 |
| 6-phosphogluconate dehydrogenase | lmo1376 | 16803416 | Energy Metabolism | Pentose Phosphate pathway | 14 | 29 | 0.000 | 0.000 | 2 | 0.000 | 0.027 | 4 |
|  | lmo1377 | 16803417 | Regulatory Functions | Other | 8 | 6 | 0.000 | 0.046 | 2 | 0.000 | 0.066 | 2 |
| hypothetical protein lmo1381 | lmo1381 | 16803421 | Fatty Acid and Phospholipid Metabolism | Other | 9 | 17 | 0.000 | 0.077 | 2 | 0.002 | 0.144 | 3 |
| CD4+ T cell-stimulating antigen, lipoprotein | lmo1388 | 16803428 | Function Unknown | Unknown General | 23 | 103 | 0.000 | 0.051 | 3 | 0.092 | 0.231 | 11 |
| hypothetical protein lmo1389 | lmo1389 | 16803429 | Transport and Binding Proteins | Carbohydrates, Organic Alcohols and Acids | 8 | 16 | 0.000 | 0.053 | 2 | 0.000 | 0.019 | 3 |
|  | lmo1396 | 16803436 | Fatty Acid and Phospholipid Metabolism | Biosynthesis | 6 | 6 | 0.076 | 0.001 | 2 | 0.101 | 0.102 | 2 |
|  | lmo1398 | 16803438 | DNA Metabolism | DNA Replication, Recombination and Repair | 10 | 10 | 0.000 | 0.066 | 2 | 0.092 | 0.117 | 2 |
|  | lmo1399 | 16803439 | Function Unknown | Unknown General | 9 | 5 | 0.003 | 0.015 | 2 | 0.092 | 0.240 | 2 |
|  | lmo1403 | 16803443 | DNA Metabolism | DNA Replication, Recombination and Repair | 7 | 7 | 0.000 | 0.215 | 2 | 0.101 | 0.003 | 2 |
|  | lmo1404 | 16803444 | DNA Metabolism | DNA Replication, Recombination and Repair | 7 | 5 | 0.000 | 0.135 | 2 | 0.000 | 0.097 | 2 |
| pyruvate formate-lyase | lmo1407 | 16803447 | Protein Fate | Protein Modification and Repair | 50 | 126 | 0.000 | 0.001 | 6 | 0.000 | 0.000 | 14 |
| hypothetical protein lmo1434 | lmo1434 | 16803474 | Multiple Roles | Multiple Roles | 6 | 16 | 0.000 | 0.146 | 2 | 0.092 | 0.000 | 3 |
|  | lmo1435 | 16803475 | Amino Acid Biosynthesis | Aspartate-Family | 5 | 7 | 0.076 | 0.002 | 2 | 0.101 | 0.213 | 2 |
|  | lmo1437 | 16803477 | Amino Acid Biosynthesis | Aspartate-Family | 8 | 6 | 0.064 | 0.000 | 2 | 0.101 | 0.232 | 2 |
|  | lmo1439 | 16803479 | Cellular Processes | Detoxification | 27 | 16 | 0.000 | 0.185 | 4 | 0.000 | 0.027 | 3 |
|  | lmo1454 | 16803494 | Transcription | Transcription Factors | 7 | 5 | 0.000 | 0.002 | 2 | 0.000 | 0.114 | 2 |
|  | lmo1458 | 16803498 | Protein Synthesis | tRNA Aminoacylation | 5 | 7 | 0.064 | 0.292 | 2 | 0.000 | 0.002 | 2 |
| GTP-binding protein Era | lmo1462 | 16803502 | Multiple Roles | Multiple Roles | 13 | 6 | 0.000 | 0.054 | 2 | 0.092 | 0.161 | 2 |
|  | lmo1463 | 16803503 | Purines, Pyrimidines, Nucleosides and Nucleotides | Salvage of Nucleotides and Nucleosides | 5 | 6 | 0.008 | 0.048 | 2 | 0.000 | 0.011 | 2 |
|  | lmo1468 | 16803508 | Function Unknown | Unknown General | 5 | 7 | 0.000 | 0.001 | 2 | 0.000 | 0.000 | 2 |
|  | lmo1472 | 16803512 | Multiple Roles | Multiple Roles | 10 | 11 | 0.064 | 0.299 | 2 | 0.000 | 0.012 | 2 |
| class I heat-shock protein DnaK | lmo1473 | 16803513 | Protein Fate | Protein Folding and Stabilisation | 41 | 84 | 0.000 | 0.000 | 5 | 0.000 | 0.004 | 9 |
| heat shock protein GrpE | lmo1474 | 16803514 | Protein Fate | Protein Folding and Stabilisation | 5 | 15 | 0.000 | 0.000 | 2 | 0.000 | 0.000 | 3 |
| hypothetical protein lmo1489 | lmo1489 | 16803529 | Function Unknown | Unknown General | 8 | 13 | 0.000 | 0.097 | 2 | 0.000 | 0.000 | 2 |
|  | lmo1491 | 16803531 | Function Unknown | Unknown General | 6 | 5 | 0.000 | 0.052 | 2 | 0.000 | 0.009 | 2 |
|  | lmo1493 | 16803533 | Protein Fate | Degradation of Proteins, Peptides and Glycopeptides | 6 | 9 | 0.076 | 0.005 | 2 | 0.000 | 0.000 | 2 |
|  | lmo1496 | 16803536 | Transcription | Transcription Factors | 6 | 9 | 0.076 | 0.005 | 2 | 0.000 | 0.244 | 2 |
|  | lmo1497 | 16803537 | Purines, Pyrimidines, Nucleosides and Nucleotides | Salvage of Nucleotides and Nucleosides | 9 | 5 | 0.000 | 0.043 | 2 | 0.000 | 0.001 | 2 |
|  | lmo1499 | 16803539 | Function Unknown | Unknown General | 7 | 6 | 0.000 | 0.000 | 2 | 0.000 | 0.000 | 2 |
|  | lmo1501 | 16803541 | Function Unknown | Unknown General | 6 | 5 | 0.000 | 0.089 | 2 | 0.101 | 0.156 | 2 |
| UPF0297 protein lmo1503 | lmo1503 | 16803543 | Function Unknown | Unknown General | 17 | 5 | 0.000 | 0.157 | 3 | 0.000 | 0.206 | 2 |
| alanyl-tRNA synthetase | lmo1504 | 16803544 | Protein Synthesis | tRNA Aminoacylation | 17 | 5 | 0.000 | 0.285 | 3 | 0.000 | 0.279 | 2 |
|  | lmo1515 | 16803555 | Function Unknown | Unknown General | 8 | 6 | 0.000 | 0.025 | 2 | 0.000 | 0.000 | 2 |
|  | lmo1519 | 16803559 | Protein Synthesis | tRNA Aminoacylation | 7 | 8 | 0.007 | 0.284 | 2 | 0.000 | 0.284 | 2 |
|  | lmo1521 | 16803561 | Cell Envelope | Biosynthesis and Degradation of Murein Sacculus and Peptidoglycan | 5 | 7 | 0.000 | 0.130 | 2 | 0.000 | 0.000 | 2 |
| RelA-like protein (Hypothetical) | lmo1523 | 16803563 | Function Unknown | Unknown General | 19 | 41 | 0.030 | 0.110 | 3 | 0.000 | 0.090 | 2 |
|  | lmo1527 | 16803567 | Cell Envelope | Other | 6 | 5 | 0.000 | 0.230 | 2 | 0.101 | 0.073 | 2 |
|  | lmo1528 | 16803568 | Function Unknown | Unknown General | 9 | 5 | 0.000 | 0.006 | 2 | 0.000 | 0.218 | 2 |
| preprotein translocase subunit YajC | lmo1529 | 16803569 | Protein Fate | Protein and Peptide secretion and Trafficking | 12 | 17 | 0.069 | 0.003 | 2 | 0.101 | 0.156 | 3 |
|  | lmo1531 | 16803571 | Protein Synthesis | Translation Factors | 7 | 5 | 0.003 | 0.191 | 2 | 0.003 | 0.118 | 2 |
| GTPase ObgE | lmo1537 | 16803577 | Multiple Roles | Multiple Roles | 19 | 6 | 0.000 | 0.100 | 3 | 0.000 | 0.160 | 4 |
|  | lmo1538 | 16803578 | Energy Metabolism | Other | 5 | 6 | 0.076 | 0.126 | 2 | 0.101 | 0.000 | 2 |
| 50S ribosomal protein L27 | lmo1540 | 16803580 | Protein Synthesis | Ribosomal Proteins: Synthesis and Modification | 14 | 9 | 0.064 | 0.184 | 2 | 0.000 | 0.021 | 2 |
|  | lmo1541 | 16803581 | Function Unknown | Unknown General | 7 | 5 | 0.000 | 0.016 | 2 | 0.000 | 0.000 | 2 |
| RL21_LISMF | lmo1542 | 16803582 | Protein Synthesis | Ribosomal Proteins: Synthesis and Modification | 78 | 6 | 0.000 | 0.203 | 2 | 0.005 | 0.001 | 9 |
|  | lmo1547 | 16803587 | Cell Envelope | Biosynthesis and Degradation of Murein Sacculus and Peptidoglycan | 8 | 7 | 0.005 | 0.021 | 2 | 0.006 | 0.183 | 2 |
|  | lmo1552 | 16803592 | Protein Synthesis | tRNA Aminoacylation | 9 | 5 | 0.005 | 0.031 | 2 | 0.002 | 0.149 | 2 |
|  | lmo1554 | 16803594 | Biosynthesis of Cofactors, Prosthetic Groups and Carriers | Haeme, Porphyrin and Cobalamin | 5 | 8 | 0.000 | 0.000 | 2 | 0.000 | 0.238 | 2 |
|  | lmo1566 | 16803606 | Energy Metabolism | TCA Cycle | 10 | 6 | 0.000 | 0.019 | 2 | 0.000 | 0.154 | 2 |
| pyruvate kinase | lmo1570 | 16803610 | Energy Metabolism | Glycolysis/Gluconeogenesis | 29 | 60 | 0.000 | 0.114 | 4 | 0.000 | 0.229 | 7 |
| 6-phosphofructokinase | lmo1571 | 16803611 | Energy Metabolism | Glycolysis/Gluconeogenesis | 36 | 44 | 0.000 | 0.244 | 5 | 0.000 | 0.000 | 5 |
|  | lmo1577 | 16803617 | Multiple Roles | Multiple Roles | 7 | 5 | 0.064 | 0.292 | 2 | 0.000 | 0.207 | 2 |
| hypothetical protein lmo1578 | lmo1578 | 16803618 | Protein Fate | Degradation of Proteins, Peptides and Glycopeptides | 12 | 7 | 0.076 | 0.000 | 2 | 0.014 | 0.010 | 2 |
|  | lmo1579 | 16803619 | Energy Metabolism | Amino Acids and Amines | 22 | 20 | 0.000 | 0.006 | 3 | 0.000 | 0.018 | 3 |
| hypothetical protein lmo1580 | lmo1580 | 16803620 | Cellular Processes | Adaptations to Atypical Conditions | 22 | 49 | 0.064 | 0.250 | 3 | 0.101 | 0.094 | 6 |
| hypothetical protein lmo1503 | lmo1583 | 16803623 | Cellular Processes | Detoxification | 17 | 45 | 0.000 | 0.052 | 3 | 0.000 | 0.004 | 6 |
|  | lmo1592 | 16803632 | Biosynthesis of Cofactors, Prosthetic Groups and Carriers | Thiamine | 5 | 6 | 0.002 | 0.149 | 2 | 0.003 | 0.115 | 2 |
| EZRA_LISMO | lmo1594 | 16803634 | Function Unknown | Unknown General | 12 | 6 | 0.000 | 0.000 | 2 | 0.000 | 0.002 | 2 |
| small subunit ribosomal protein S4 | lmo1596 | 16803636 | Protein Synthesis | Ribosomal Proteins: Synthesis and Modification | 20 | 5 | 0.000 | 0.078 | 2 | 0.064 | 0.226 | 3 |
| catabolite control protein A | lmo1599 | 16803639 | Regulatory Functions | Other | 5 | 20 | 0.069 | 0.005 | 2 | 0.000 | 0.215 | 3 |
|  | lmo1600 | 16803640 | Amino Acid Biosynthesis | Aromatic Amino Acid | 5 | 6 | 0.000 | 0.010 | 2 | 0.000 | 0.022 | 2 |
| hypothetical protein lmo1601 | lmo1601 | 16803641 | Cellular Processes | Adaptations to Atypical Conditions | 13 | 66 | 0.006 | 0.020 | 2 | 0.007 | 0.225 | 8 |
|  | lmo1602 | 16803642 | Function Unknown | Unknown General | 5 | 7 | 0.076 | 0.278 | 2 | 0.101 | 0.190 | 2 |
|  | lmo1603 | 16803643 | Protein Fate | Degradation of Proteins, Peptides and Glycopeptides | 8 | 10 | 0.000 | 0.002 | 2 | 0.101 | 0.166 | 2 |
|  | lmo1604 | 16803644 | Multiple Roles | Multiple Roles | 8 | 9 | 0.000 | 0.285 | 2 | 0.101 | 0.256 | 2 |
|  | lmo1606 | 16803646 | Function Unknown | Unknown General | 5 | 6 | 0.000 | 0.242 | 2 | 0.092 | 0.248 | 2 |
| hypothetical protein lmo1611 | lmo1611 | 16803651 | Protein Fate | Degradation of Proteins, Peptides and Glycopeptides | 20 | 14 | 0.076 | 0.001 | 3 | 0.000 | 0.184 | 2 |
| hypothetical protein lmo1612 | lmo1612 | 16803652 | Function Unknown | Unknown General | 19 | 39 | 0.000 | 0.248 | 3 | 0.092 | 0.014 | 5 |
|  | lmo1619 | 16803659 | Energy Metabolism | Amino Acids and Amines | 7 | 5 | 0.002 | 0.155 | 2 | 0.002 | 0.055 | 2 |
| dipeptidase PepV | lmo1620 | 16803660 | Protein Fate | Degradation of Proteins, Peptides and Glycopeptides | 24 | 19 | 0.069 | 0.099 | 3 | 0.000 | 0.089 | 3 |
|  | lmo1631 | 16803671 | Amino Acid Biosynthesis | Aromatic Amino Acid | 5 | 6 | 0.000 | 0.002 | 2 | 0.000 | 0.000 | 2 |
| bifunctional acetaldehyde-CoA | lmo1634 | 16803674 | Energy Metabolism | Fermentation | 72 | 149 | 0.000 | 0.000 | 8 | 0.000 | 0.002 | 16 |
|  | lmo1636 | 16803676 | Transport and Binding Proteins | Unknown Substrate | 7 | 5 | 0.000 | 0.146 | 2 | 0.011 | 0.152 | 2 |
|  | lmo1643 | 16803683 | Function Unknown | Unknown General | 5 | 8 | 0.000 | 0.147 | 2 | 0.000 | 0.146 | 2 |
| 1-acyl-sn-glycerol-3-phosphate acyltransferase | lmo1647 | 16803687 | Multiple Roles | Multiple Roles | 5 | 13 | 0.007 | 0.284 | 2 | 0.092 | 0.239 | 2 |
|  | lmo1649 | 16803689 | Function Unknown | Unknown General | 5 | 8 | 0.000 | 0.223 | 2 | 0.000 | 0.191 | 2 |
|  | lmo1656 | 16803696 | Function Unknown | Unknown General | 5 | 6 | 0.008 | 0.029 | 2 | 0.011 | 0.230 | 2 |
| elongation factor Ts | lmo1657 | 16803697 | Protein Synthesis | Other | 13 | 9 | 0.008 | 0.236 | 2 | 0.000 | 0.001 | 2 |
|  | lmo1658 | 16803698 | Protein Synthesis | Ribosomal Proteins: Synthesis and Modification | 46 | 45 | 0.064 | 0.162 | 6 | 0.000 | 0.262 | 6 |
|  | lmo1660 | 16803700 | Protein Synthesis | tRNA Aminoacylation | 9 | 6 | 0.064 | 0.103 | 2 | 0.000 | 0.158 | 2 |
|  | lmo1663 | 16803703 | Function Unknown | Unknown General | 5 | 7 | 0.000 | 0.003 | 2 | 0.000 | 0.000 | 2 |
| hypothetical protein lmo1668 | lmo1668 | 16803708 | Function Unknown | Unknown General | 10 | 31 | 0.003 | 0.095 | 2 | 0.004 | 0.135 | 4 |
| zinc transport system substrate-binding protein | lmo1671 | 16803711 | Multiple Roles | Multiple Roles | 5 | 9 | 0.003 | 0.191 | 2 | 0.092 | 0.195 | 2 |
|  | lmo1673 | 16803713 | Biosynthesis of Cofactors, Prosthetic Groups and Carriers | Menaquinone and Ubiquinone | 7 | 8 | 0.000 | 0.294 | 2 | 0.000 | 0.003 | 2 |
| Hypothetical protein | lmo1692 | 16803732 | Function Unknown | Unknown General | 9 | 13 | 0.069 | 0.227 | 2 | 0.092 | 0.001 | 2 |
| methionine aminopeptidase | lmo1709 | 16803749 | Protein Fate | Protein Modification and Repair | 11 | 21 | 0.000 | 0.002 | 2 | 0.001 | 0.000 | 3 |
|  | lmo1711 | 16803751 | Protein Fate | Degradation of Proteins, Peptides and Glycopeptides | 6 | 5 | 0.069 | 0.186 | 2 | 0.101 | 0.236 | 2 |
|  | lmo1718 | 16803758 | Function Unknown | Unknown General | 12 | 14 | 0.069 | 0.000 | 2 | 0.092 | 0.089 | 2 |
| PTS system, cellobiose-specific IIA component | lmo1719 | 16803759 | Multiple Roles | Multiple Roles | 5 | 15 | 0.064 | 0.184 | 2 | 0.092 | 0.242 | 3 |
|  | lmo1721 | 16803761 | Regulatory Functions | Other | 7 | 6 | 0.000 | 0.012 | 2 | 0.000 | 0.135 | 2 |
|  | lmo1737 | 16803777 | Central Intermediary Metabolism | Other | 5 | 7 | 0.076 | 0.034 | 2 | 0.101 | 0.223 | 2 |
|  | lmo1750 | 16803790 | Function Unknown | Unknown General | 7 | 5 | 0.000 | 0.003 | 2 | 0.000 | 0.001 | 2 |
|  | lmo1754 | 16803794 | Protein Synthesis | tRNA Aminoacylation | 8 | 9 | 0.000 | 0.000 | 2 | 0.000 | 0.191 | 2 |
| glutamyl-tRNA(Gln) amidotransferase | lmo1755 | 16803795 | Protein Synthesis | tRNA Aminoacylation | 17 | 8 | 0.000 | 0.046 | 2 | 0.000 | 0.102 | 2 |
| aspartyl/glutamyl-tRNA amidotransferase subunit C | lmo1756 | 16803796 | Protein Synthesis | tRNA Aminoacylation | 51 | 15 | 0.069 | 0.005 | 3 | 0.000 | 0.000 | 6 |
|  | lmo1757 | 16803797 | Function Unknown | Unknown General | 5 | 7 | 0.003 | 0.002 | 2 | 0.003 | 0.000 | 2 |
|  | lmo1758 | 16803798 | DNA Metabolism | DNA Replication, Recombination and Repair | 6 | 5 | 0.000 | 0.055 | 2 | 0.000 | 0.000 | 2 |
|  | lmo1763 | 16803803 | Function Unknown | Unknown General | 5 | 7 | 0.000 | 0.179 | 2 | 0.000 | 0.144 | 2 |
|  | lmo1771 | 16803811 | Purines, Pyrimidines, Nucleosides and Nucleotides | Purine Ribonucleotide Biosynthesis | 5 | 6 | 0.000 | 0.299 | 2 | 0.000 | 0.051 | 2 |
|  | lmo1773 | 16803813 | Purines, Pyrimidines, Nucleosides and Nucleotides | Purine Ribonucleotide Biosynthesis | 6 | 5 | 0.000 | 0.146 | 2 | 0.000 | 0.062 | 2 |
|  | lmo1780 | 16803820 | Protein Fate | Degradation of Proteins, Peptides and Glycopeptides | 8 | 8 | 0.000 | 0.017 | 2 | 0.000 | 0.096 | 2 |
|  | lmo1785 | 16803825 | Protein Synthesis | Other | 11 | 14 | 0.000 | 0.012 | 2 | 0.000 | 0.245 | 2 |
| 50S ribosomal protein L19 | lmo1787 | 16803827 | Protein Synthesis | Ribosomal Proteins: Synthesis and Modification | 19 | 14 | 0.064 | 0.241 | 2 | 0.000 | 0.294 | 3 |
|  | lmo1791 | 16803831 | Function Unknown | Unknown General | 15 | 11 | 0.000 | 0.104 | 3 | 0.000 | 0.000 | 2 |
|  | lmo1793 | 16803833 | Transcription | RNA Processing | 7 | 7 | 0.000 | 0.016 | 2 | 0.000 | 0.004 | 2 |
|  | lmo1794 | 16803834 | Function Unknown | Unknown General | 6 | 5 | 0.000 | 0.110 | 2 | 0.101 | 0.254 | 2 |
| 30S ribosomal protein S16 | lmo1797 | 16803837 | Protein Synthesis | Ribosomal Proteins: Synthesis and Modification | 48 | 5 | 0.000 | 0.108 | 2 | 0.000 | 0.266 | 6 |
|  | lmo1804 | 16803844 | Cellular Processes | Cell Division | 5 | 6 | 0.000 | 0.011 | 2 | 0.000 | 0.000 | 2 |
|  | lmo1806 | 16803846 | Fatty Acid and Phospholipid Metabolism | Biosynthesis | 8 | 5 | 0.006 | 0.232 | 2 | 0.007 | 0.225 | 2 |
| hypothetical protein lmo1807 | lmo1807 | 16803847 | Fatty Acid and Phospholipid Metabolism | Biosynthesis | 8 | 14 | 0.000 | 0.068 | 2 | 0.101 | 0.002 | 2 |
|  | lmo1808 | 16803848 | Fatty Acid and Phospholipid Metabolism | Biosynthesis | 7 | 8 | 0.000 | 0.009 | 2 | 0.101 | 0.228 | 2 |
|  | lmo1813 | 16803853 | Energy Metabolism | Amino Acids and Amines | 12 | 8 | 0.069 | 0.065 | 2 | 0.092 | 0.200 | 2 |
|  | lmo1814 | 16803854 | Function Unknown | Unknown General | 7 | 8 | 0.000 | 0.008 | 2 | 0.001 | 0.143 | 2 |
|  | lmo1820 | 16803860 | Regulatory Functions | Other | 6 | 5 | 0.064 | 0.241 | 2 | 0.000 | 0.055 | 2 |
| hypothetical protein lmo1826 | lmo1826 | 16803866 | Function Unknown | Unknown General | 8 | 17 | 0.000 | 0.170 | 2 | 0.000 | 0.000 | 3 |
|  | lmo1829 | 16803869 | Cell Envelope | Surface Structures | 6 | 5 | 0.009 | 0.261 | 2 | 0.000 | 0.225 | 2 |
|  | lmo1835 | 16803875 | Purines, Pyrimidines, Nucleosides and Nucleotides | Pyrimidine Ribonucleotide Biosynthesis | 5 | 6 | 0.000 | 0.207 | 2 | 0.000 | 0.000 | 2 |
|  | lmo1850 | 16803890 | Regulatory Functions | Other | 5 | 6 | 0.000 | 0.108 | 2 | 0.000 | 0.014 | 2 |
|  | lmo1852 | 16803892 | Transport and Binding Proteins | Cations and Iron Carrying Compounds | 5 | 6 | 0.008 | 0.048 | 2 | 0.076 | 0.015 | 2 |
| purine nucleoside phosphorylase | lmo1856 | 16803896 | Purines, Pyrimidines, Nucleosides and Nucleotides | Salvage of Nucleotides and Nucleosides | 10 | 20 | 0.000 | 0.252 | 2 | 0.000 | 0.004 | 3 |
|  | lmo1858 | 16803898 | Multiple Roles | Multiple Roles | 7 | 6 | 0.000 | 0.203 | 2 | 0.101 | 0.081 | 2 |
|  | lmo1861 | 16803901 | Function Unknown | Unknown General | 5 | 7 | 0.076 | 0.252 | 2 | 0.101 | 0.248 | 2 |
|  | lmo1862 | 16803902 | Function Unknown | Unknown General | 5 | 6 | 0.000 | 0.017 | 2 | 0.000 | 0.000 | 2 |
|  | lmo1866 | 16803906 | Function Unknown | Unknown General | 6 | 5 | 0.000 | 0.192 | 2 | 0.000 | 0.000 | 2 |
|  | lmo1873 | 16803913 | Biosynthesis of Cofactors, Prosthetic Groups and Carriers | Folate | 6 | 5 | 0.000 | 0.021 | 2 | 0.000 | 0.000 | 2 |
|  | lmo1874 | 16803914 | Purines, Pyrimidines, Nucleosides and Nucleotides | 2'-Deoxyribonucleotide Metabolism | 5 | 8 | 0.076 | 0.219 | 2 | 0.000 | 0.001 | 2 |
|  | lmo1877 | 16803916 | Multiple Roles | Multiple Roles | 11 | 12 | 0.005 | 0.031 | 2 | 0.101 | 0.000 | 2 |
|  | lmo1878 | 16803917 | Regulatory Functions | Other | 9 | 9 | 0.000 | 0.286 | 2 | 0.000 | 0.005 | 2 |
| hypothetical protein lmo1879 | lmo1879 | 16803918 | Cellular Processes | Adaptations to Atypical Conditions | 17 | 62 | 0.000 | 0.221 | 3 | 0.101 | 0.256 | 7 |
|  | lmo1888 | 16803927 | Cellular Processes | Cell Division | 20 | 21 | 0.076 | 0.295 | 3 | 0.101 | 0.206 | 3 |
|  | lmo1892 | 16803931 | Cell Envelope | Biosynthesis and Degradation of Murein Sacculus and Peptidoglycan | 6 | 8 | 0.000 | 0.003 | 2 | 0.000 | 0.245 | 2 |
|  | lmo1896 | 16803935 | Protein Synthesis | tRNA Aminoacylation | 11 | 15 | 0.000 | 0.286 | 2 | 0.076 | 0.227 | 3 |
| 3-methyl-2-oxobutanoate hydroxymethyltransferase | lmo1902 | 16803941 | Biosynthesis of Cofactors, Prosthetic Groups and Carriers | Pantothenate and Coenzyme A | 16 | 8 | 0.000 | 0.266 | 3 | 0.092 | 0.109 | 2 |
|  | lmo1907 | 16803946 | Amino Acid Biosynthesis | Aspartate-Family | 9 | 5 | 0.076 | 0.212 | 2 | 0.101 | 0.036 | 2 |
|  | lmo1918 | 16803957 | Function Unknown | Unknown General | 6 | 5 | 0.000 | 0.014 | 2 | 0.000 | 0.001 | 2 |
|  | lmo1922 | 16803961 | Function Unknown | Unknown General | 6 | 5 | 0.000 | 0.234 | 2 | 0.000 | 0.000 | 2 |
| hypothetical protein lmo1934 | lmo1934 | 16803973 | Function Unknown | Unknown General | 566 | 1596 | 0.000 | 0.000 | 18 | 0.000 | 0.026 | 11 |
|  | lmo1936 | 16803975 | Energy Metabolism | Other | 5 | 7 | 0.000 | 0.071 | 2 | 0.000 | 0.000 | 2 |
| 30S ribosomal protein S1 | lmo1938 | 16803977 | Protein Synthesis | Ribosomal Proteins: Synthesis and Modification | 43 | 34 | 0.000 | 0.028 | 4 | 0.000 | 0.006 | 5 |
|  | lmo1946 | 16803985 | Fatty Acid and Phospholipid Metabolism | Degradation | 5 | 6 | 0.000 | 0.158 | 2 | 0.101 | 0.184 | 2 |
| hypothetical protein lmo1948 | lmo1948 | 16803987 | Regulatory Functions | Other | 12 | 5 | 0.000 | 0.028 | 2 | 0.069 | 0.246 | 2 |
| phosphopentomutase | lmo1954 | 16803993 | Purines, Pyrimidines, Nucleosides and Nucleotides | Salvage of Nucleotides and Nucleosides | 15 | 24 | 0.000 | 0.030 | 3 | 0.064 | 0.196 | 3 |
|  | lmo1956 | 16803995 | Regulatory Functions | Other | 6 | 6 | 0.000 | 0.003 | 2 | 0.069 | 0.003 | 2 |
|  | lmo1967 | 16804006 | Cellular Processes | Toxin Production and Resistance | 9 | 13 | 0.064 | 0.196 | 2 | 0.002 | 0.191 | 2 |
| acetolactate synthase | lmo1984 | 16804023 | Amino Acid Biosynthesis | Pyruvate Family | 57 | 109 | 0.000 | 0.014 | 7 | 0.000 | 0.079 | 12 |
| hypothetical protein lmo1992 | lmo1992 | 16804031 | Energy Metabolism | Fermentation | 49 | 21 | 0.007 | 0.298 | 2 | 0.009 | 0.251 | 2 |
|  | lmo1993 | 16804032 | Purines, Pyrimidines, Nucleosides and Nucleotides | Salvage of Nucleotides and Nucleosides | 11 | 10 | 0.000 | 0.185 | 2 | 0.076 | 0.000 | 2 |
|  | lmo1995 | 16804034 | Multiple Roles | Multiple Roles | 6 | 9 | 0.000 | 0.078 | 2 | 0.001 | 0.160 | 2 |
|  | lmo1998 | 16804037 | Function Unknown | Unknown General | 5 | 7 | 0.000 | 0.238 | 2 | 0.000 | 0.039 | 2 |
|  | lmo2004 | 16804043 | Regulatory Functions | Other | 6 | 5 | 0.001 | 0.173 | 2 | 0.000 | 0.134 | 2 |
| Acetolactate synthase | lmo2006 | 16804045 | Amino Acid Biosynthesis | Branched chain amino acids | 12 | 25 | 0.000 | 0.110 | 5 | 0.000 | 0.000 | 4 |
|  | lmo2007 | 16804046 | Transport and Binding Proteins | Unknown Substrate | 6 | 5 | 0.000 | 0.043 | 2 | 0.000 | 0.010 | 2 |
| hypothetical protein lmo2016 | lmo2016 | 16804055 | Cellular Processes | Adaptations to Atypical Conditions | 664 | 1178 | 0.069 | 0.058 | 17 | 0.092 | 0.238 | 29 |
| isoleucyl-tRNA synthetase | lmo2019 | 16804058 | Protein Synthesis | tRNA Aminoacylation | 12 | 6 | 0.000 | 0.003 | 2 | 0.000 | 0.004 | 2 |
| hypothetical protein lmo2020 | lmo2020 | 16804059 | Cellular Processes | Cell Division | 24 | 50 | 0.000 | 0.051 | 3 | 0.000 | 0.000 | 6 |
|  | lmo2029 | 16804068 | Function Unknown | Unknown General | 6 | 5 | 0.069 | 0.279 | 2 | 0.092 | 0.255 | 2 |
|  | lmo2030 | 16804069 | Function Unknown | Unknown General | 5 | 7 | 0.000 | 0.275 | 2 | 0.011 | 0.116 | 2 |
|  | lmo2031 | 16804070 | Function Unknown | Unknown General | 7 | 5 | 0.000 | 0.017 | 2 | 0.092 | 0.248 | 2 |
| cell division protein FtsZ | lmo2032 | 16804071 | Cellular Processes | Cell Division | 11 | 6 | 0.000 | 0.062 | 2 | 0.000 | 0.009 | 2 |
|  | lmo2033 | 16804072 | Cellular Processes | Cell Division | 5 | 8 | 0.000 | 0.000 | 2 | 0.000 | 0.000 | 2 |
|  | lmo2035 | 16804074 | Cell Envelope | Biosynthesis and Degradation of Murein Sacculus and Peptidoglycan | 5 | 7 | 0.000 | 0.203 | 2 | 0.000 | 0.255 | 2 |
|  | lmo2036 | 16804075 | Cell Envelope | Biosynthesis and Degradation of Murein Sacculus and Peptidoglycan | 5 | 7 | 0.000 | 0.281 | 2 | 0.092 | 0.208 | 2 |
|  | lmo2038 | 16804077 | Cell Envelope | Biosynthesis and Degradation of Murein Sacculus and Peptidoglycan | 7 | 5 | 0.000 | 0.290 | 2 | 0.000 | 0.000 | 2 |
|  | lmo2039 | 16804078 | Cell Envelope | Biosynthesis and Degradation of Murein Sacculus and Peptidoglycan | 6 | 6 | 0.000 | 0.246 | 2 | 0.101 | 0.141 | 2 |
|  | lmo2041 | 16804080 | Function Unknown | Unknown General | 8 | 5 | 0.076 | 0.243 | 2 | 0.101 | 0.156 | 2 |
|  | lmo2051 | 16804090 | Function Unknown | Unknown General | 7 | 5 | 0.000 | 0.002 | 2 | 0.000 | 0.000 | 2 |
|  | lmo2052 | 16804091 | Cell Envelope | Biosynthesis and Degradation of Surface Polysaccharides and Lipopolysaccharides | 8 | 5 | 0.000 | 0.004 | 2 | 0.000 | 0.000 | 2 |
| chaperonin GroEL | lmo2068 | 16804107 | Protein Fate | Protein Folding and Stabilisation | 36 | 58 | 0.000 | 0.001 | 5 | 0.101 | 0.017 | 7 |
| co-chaperonin GroES | lmo2069 | 16804108 | Protein Fate | Protein Folding and Stabilisation | 31 | 425 | 0.000 | 0.076 | 4 | 0.000 | 0.055 | 24 |
| redox-sensing transcriptional repressor Rex | lmo2072 | 16804111 | Viral Functions | General | 13 | 5 | 0.000 | 0.001 | 2 | 0.076 | 0.126 | 2 |
|  | lmo2079 | 16804118 | Purines, Pyrimidines, Nucleosides and Nucleotides | 2'-Deoxyribonucleotide Metabolism | 7 | 9 | 0.010 | 0.222 | 2 | 0.090 | 0.211 | 3 |
| hypothetical protein lmo2089 | lmo2089 | 16804128 | Fatty Acid and Phospholipid Metabolism | Degradation | 9 | 20 | 0.000 | 0.152 | 2 | 0.092 | 0.000 | 3 |
|  | lmo2094 | 16804133 | Energy Metabolism | Sugars | 7 | 8 | 0.000 | 0.139 | 2 | 0.000 | 0.000 | 2 |
|  | lmo2097 | 16804136 | Multiple Roles | Multiple Roles | 5 | 7 | 0.008 | 0.236 | 2 | 0.021 | 0.042 | 2 |
| pyridoxine biosynthesis protein | lmo2101 | 16804140 | Biosynthesis of Cofactors, Prosthetic Groups and Carriers | Pyridoxine | 12 | 7 | 0.076 | 0.227 | 2 | 0.101 | 0.241 | 2 |
|  | lmo2103 | 16804142 | Multiple Roles | Multiple Roles | 8 | 5 | 0.064 | 0.162 | 2 | 0.006 | 0.241 | 2 |
|  | lmo2110 | 16804149 | Energy Metabolism | Sugars | 7 | 5 | 0.004 | 0.019 | 2 | 0.005 | 0.000 | 2 |
|  | lmo2111 | 16804150 | Multiple Roles | Multiple Roles | 7 | 5 | 0.064 | 0.103 | 2 | 0.000 | 0.001 | 2 |
|  | lmo2113 | 16804152 | Function Unknown | Unknown General | 9 | 5 | 0.000 | 0.072 | 2 | 0.000 | 0.000 | 2 |
| hypothetical protein lmo2114 | lmo2114 | 16804153 | Transport and Binding Proteins | Unknown Substrate | 10 | 5 | 0.000 | 0.299 | 2 | 0.000 | 0.000 | 2 |
|  | lmo2118 | 16804157 | Function Unknown | Unknown General | 6 | 6 | 0.000 | 0.286 | 2 | 0.000 | 0.009 | 2 |
|  | lmo2120 | 16804159 | Function Unknown | Unknown General | 5 | 8 | 0.000 | 0.028 | 2 | 0.000 | 0.000 | 2 |
|  | lmo2155 | 16804194 | Purines, Pyrimidines, Nucleosides and Nucleotides | 2'-Deoxyribonucleotide Metabolism | 5 | 9 | 0.004 | 0.271 | 2 | 0.000 | 0.070 | 2 |
| hypothetical protein lmo2188 | lmo2188 | 16804227 | Protein Fate | Degradation of Proteins, Peptides and Glycopeptides | 11 | 6 | 0.000 | 0.160 | 2 | 0.092 | 0.235 | 2 |
|  | lmo2191 | 16804230 | Transcription | Other | 7 | 7 | 0.000 | 0.000 | 2 | 0.000 | 0.000 | 2 |
|  | lmo2192 | 16804231 | Transport and Binding Proteins | Amino Acids, Peptides and Amines | 8 | 5 | 0.069 | 0.099 | 2 | 0.000 | 0.183 | 2 |
| hypothetical protein lmo2193 | lmo2193 | 16804232 | Transport and Binding Proteins | Amino Acids, Peptides and Amines | 6 | 11 | 0.069 | 0.186 | 2 | 0.066 | 0.210 | 2 |
|  | lmo2195 | 16804234 | Transport and Binding Proteins | Amino Acids, Peptides and Amines | 5 | 7 | 0.000 | 0.017 | 2 | 0.066 | 0.006 | 2 |
| hypothetical protein lmo2196 | lmo2196 | 16804235 | Multiple Roles | Multiple Roles | 46 | 105 | 0.000 | 0.000 | 6 | 0.000 | 0.000 | 12 |
|  | lmo2201 | 16804240 | Fatty Acid and Phospholipid Metabolism | Biosynthesis | 12 | 13 | 0.000 | 0.043 | 2 | 0.001 | 0.006 | 2 |
| 3-oxoacyl-(acyl carrier protein) synthase III | lmo2202 | 16804241 | Fatty Acid and Phospholipid Metabolism | Biosynthesis | 11 | 5 | 0.000 | 0.042 | 2 | 0.000 | 0.256 | 2 |
| phosphoglyceromutase | lmo2205 | 16804244 | Energy Metabolism | Glycolysis/Gluconeogenesis | 49 | 5 | 0.000 | 0.004 | 6 | 0.000 | 0.059 | 2 |
| hypothetical protein lmo2206 | lmo2206 | 16804245 | Protein Fate | Degradation of Proteins, Peptides and Glycopeptides | 25 | 7 | 0.069 | 0.152 | 4 | 0.000 | 0.086 | 2 |
|  | lmo2211 | 16804250 | Biosynthesis of Cofactors, Prosthetic Groups and Carriers | Haeme, Porphyrin and Cobalamin | 5 | 6 | 0.000 | 0.245 | 2 | 0.101 | 0.087 | 2 |
|  | lmo2214 | 16804253 | Function Unknown | Unknown General | 6 | 7 | 0.000 | 0.003 | 2 | 0.000 | 0.016 | 2 |
|  | lmo2216 | 16804255 | Function Unknown | Unknown General | 5 | 7 | 0.001 | 0.173 | 2 | 0.001 | 0.000 | 2 |
| hypothetical protein lmo2217 | lmo2217 | 16804256 | Cellular Processes | Adaptations to Atypical Conditions | 8 | 123 | 0.069 | 0.000 | 2 | 0.092 | 0.015 | 13 |
|  | lmo2219 | 16804258 | Viral Functions | General | 8 | 11 | 0.000 | 0.153 | 2 | 0.000 | 0.017 | 2 |
| 3'-5' exoribonuclease YhaM | lmo2220 | 16804259 | Function Unknown | Unknown General | 10 | 5 | 0.000 | 0.072 | 2 | 0.000 | 0.000 | 2 |
|  | lmo2221 | 16804260 | Function Unknown | Unknown General | 6 | 5 | 0.000 | 0.001 | 2 | 0.000 | 0.001 | 2 |
| hypothetical protein lmo2223 | lmo2223 | 16804262 | Function Unknown | Unknown General | 12 | 29 | 0.000 | 0.008 | 2 | 0.000 | 0.000 | 4 |
|  | lmo2229 | 16804268 | Cell Envelope | Biosynthesis and Degradation of Murein Sacculus and Peptidoglycan | 5 | 6 | 0.000 | 0.016 | 2 | 0.000 | 0.002 | 2 |
|  | lmo2235 | 16804274 | Cellular Processes | Detoxification | 5 | 6 | 0.000 | 0.009 | 2 | 0.010 | 0.000 | 2 |
|  | lmo2248 | 16804287 | Function Unknown | Unknown General | 11 | 8 | 0.010 | 0.089 | 2 | 0.014 | 0.010 | 2 |
|  | lmo2256 | 16804295 | Biosynthesis of Cofactors, Prosthetic Groups and Carriers | Thiamine | 9 | 11 | 0.000 | 0.001 | 2 | 0.000 | 0.239 | 2 |
|  | lmo2258 | 16804297 | Function Unknown | Unknown General | 7 | 5 | 0.076 | 0.258 | 2 | 0.101 | 0.166 | 2 |
|  | lmo2267 | 16804306 | DNA Metabolism | Degradation of DNA | 7 | 10 | 0.069 | 0.246 | 2 | 0.092 | 0.121 | 2 |
|  | lmo2308 | 16804347 | DNA Metabolism | DNA Replication, Recombination and Repair | 9 | 10 | 0.000 | 0.014 | 2 | 0.000 | 0.155 | 2 |
| PTS system, fructose-specific IIA component | lmo2335 | 16804373 | Multiple Roles | Multiple Roles | 5 | 12 | 0.000 | 0.046 | 2 | 0.000 | 0.001 | 2 |
|  | lmo2337 | 16804375 | Regulatory Functions | Other | 5 | 6 | 0.000 | 0.072 | 2 | 0.000 | 0.047 | 2 |
|  | lmo2340 | 16804378 | Function Unknown | Unknown General | 5 | 7 | 0.000 | 0.000 | 2 | 0.000 | 0.184 | 2 |
| hypothetical protein | lmo2362 | 16804400 | Transport and Binding Proteins | Amino Acids, Peptides and Amines | 25 | 42 | 0.000 | 0.160 | 4 | 0.000 | 0.198 | 5 |
| hypothetical protein lmo2363 | lmo2363 | 16804401 | Multiple Roles | Multiple Roles | 54 | 5 | 0.069 | 0.005 | 6 | 0.092 | 0.165 | 2 |
| glucose-6-phosphate isomerase | lmo2367 | 16804405 | Energy Metabolism | Glycolysis/Gluconeogenesis | 37 | 61 | 0.000 | 0.000 | 5 | 0.000 | 0.190 | 7 |
| general stress protein 13 | lmo2369 | 16804407 | Multiple Roles | Multiple Roles | 17 | 39 | 0.000 | 0.012 | 3 | 0.000 | 0.005 | 5 |
| hypothetical protein lmo2372 | lmo2372 | 16804410 | Transport and Binding Proteins | Unknown Substrate | 10 | 5 | 0.076 | 0.219 | 2 | 0.002 | 0.155 | 2 |
|  | lmo2373 | 16804411 | Multiple Roles | Multiple Roles | 5 | 7 | 0.064 | 0.241 | 2 | 0.092 | 0.195 | 2 |
|  | lmo2376 | 16804414 | Protein Fate | Protein Folding and Stabilisation | 6 | 9 | 0.000 | 0.125 | 2 | 0.000 | 0.241 | 2 |
| NADH Dehydrogenase | lmo2389 | 16804427 | Central Intermediary Metabolism | Other | 12 | 7 | 0.000 | 0.255 | 2 | 0.092 | 0.001 | 2 |
|  | lmo2391 | 16804429 | Function Unknown | Unknown General | 6 | 5 | 0.000 | 0.102 | 2 | 0.000 | 0.089 | 2 |
|  | lmo2396 | 16804434 | Transport and Binding Proteins | Amino Acids, Peptides and Amines | 6 | 7 | 0.000 | 0.000 | 2 | 0.000 | 0.039 | 2 |
|  | lmo2406 | 16804444 | Function Unknown | Unknown General | 9 | 5 | 0.076 | 0.071 | 2 | 0.101 | 0.236 | 2 |
|  | lmo2411 | 16804449 | Function Unknown | Unknown General | 8 | 5 | 0.000 | 0.000 | 2 | 0.000 | 0.096 | 2 |
|  | lmo2414 | 16804452 | Function Unknown | Unknown General | 9 | 13 | 0.069 | 0.003 | 2 | 0.092 | 0.235 | 2 |
|  | lmo2415 | 16804453 | Transport and Binding Proteins | Unknown Substrate | 6 | 5 | 0.000 | 0.185 | 2 | 0.007 | 0.298 | 2 |
| hypothetical protein lmo2417 | lmo2417 | 16804455 | Function Unknown | Unknown General | 15 | 6 | 0.000 | 0.003 | 3 | 0.000 | 0.086 | 2 |
| hypothetical protein lmo2421 | lmo2421 | 16804459 | Regulatory Functions | Other | 8 | 13 | 0.000 | 0.001 | 2 | 0.000 | 0.002 | 2 |
|  | lmo2425 | 16804463 | Energy Metabolism | Amino Acids and Amines | 6 | 6 | 0.011 | 0.152 | 2 | 0.014 | 0.240 | 2 |
|  | lmo2426 | 16804464 | Multiple Roles | Multiple Roles | 14 | 13 | 0.000 | 0.108 | 2 | 0.101 | 0.240 | 2 |
|  | lmo2436 | 16804474 | Energy Metabolism | Sugars | 13 | 17 | 0.000 | 0.040 | 2 | 0.000 | 0.096 | 3 |
|  | lmo2437 | 16804475 | Multiple Roles | Multiple Roles | 6 | 5 | 0.066 | 0.195 | 2 | 0.009 | 0.065 | 2 |
|  | lmo2455 | 16804493 | Energy Metabolism | Glycolysis/Gluconeogenesis | 126 | 127 | 0.066 | 0.210 | 14 | 0.092 | 0.256 | 14 |
| phosphoglyceromutase | lmo2456 | 16804494 | Energy Metabolism | Glycolysis/Gluconeogenesis | 49 | 33 | 0.066 | 0.006 | 6 | 0.092 | 0.244 | 4 |
| triosephosphate isomerase | lmo2457 | 16804495 | Energy Metabolism | Glycolysis/Gluconeogenesis | 22 | 34 | 0.000 | 0.079 | 3 | 0.101 | 0.076 | 4 |
|  | lmo2459 | 16804497 | Energy Metabolism | Glycolysis/Gluconeogenesis | 54 | 60 | 0.064 | 0.001 | 6 | 0.101 | 0.220 | 7 |
| ATP-dependent Clp protease proteolytic subunit | lmo2468 | 16804506 | Protein Fate | Degradation of Proteins, Peptides and Glycopeptides | 12 | 6 | 0.076 | 0.015 | 2 | 0.000 | 0.065 | 2 |
|  | lmo2473 | 16804511 | Function Unknown | Unknown General | 6 | 5 | 0.000 | 0.000 | 2 | 0.000 | 0.065 | 2 |
|  | lmo2474 | 16804512 | Function Unknown | Unknown General | 7 | 8 | 0.000 | 0.023 | 2 | 0.000 | 0.000 | 2 |
| phosphoglucomutase | lmo2475 | 16804513 | Energy Metabolism | Sugars | 5 | 20 | 0.000 | 0.000 | 2 | 0.001 | 0.240 | 3 |
|  | lmo2477 | 16804515 | Energy Metabolism | Sugars | 6 | 5 | 0.000 | 0.000 | 2 | 0.000 | 0.000 | 2 |
| hypothetical protein lmo2487 | lmo2487 | 16804525 | Energy Metabolism | Electron Transport | 19 | 10 | 0.069 | 0.033 | 3 | 0.092 | 0.222 | 2 |
|  | lmo2488 | 16804526 | DNA Metabolism | DNA Replication, Recombination and Repair | 7 | 6 | 0.000 | 0.005 | 2 | 0.000 | 0.000 | 2 |
|  | lmo2491 | 16804529 | Function Unknown | Unknown General | 8 | 6 | 0.000 | 0.224 | 2 | 0.000 | 0.004 | 2 |
|  | lmo2493 | 16804531 | Regulatory Functions | Other | 10 | 13 | 0.000 | 0.008 | 2 | 0.003 | 0.027 | 2 |
|  | lmo2505 | 16804543 | Multiple Roles | Multiple Roles | 7 | 5 | 0.006 | 0.133 | 2 | 0.101 | 0.174 | 2 |
|  | lmo2506 | 16804544 | Cellular Processes | Cell Division | 5 | 6 | 0.000 | 0.004 | 2 | 0.000 | 0.042 | 2 |
| preprotein translocase subunit SecA | lmo2510 | 16804548 | Protein Fate | Protein and Peptide secretion and Trafficking | 6 | 11 | 0.000 | 0.003 | 2 | 0.000 | 0.000 | 2 |
| hypothetical protein lmo2511 | lmo2511 | 16804549 | Protein Synthesis | Ribosomal Proteins: Synthesis and Modification | 76 | 14 | 0.001 | 0.173 | 2 | 0.000 | 0.094 | 9 |
|  | lmo2524 | 16804562 | Fatty Acid and Phospholipid Metabolism | Biosynthesis | 7 | 5 | 0.000 | 0.244 | 2 | 0.014 | 0.000 | 2 |
|  | lmo2525 | 16804563 | Cell Envelope | Biosynthesis and Degradation of Murein Sacculus and Peptidoglycan | 7 | 10 | 0.006 | 0.104 | 2 | 0.009 | 0.127 | 2 |
| F0F1 ATP synthase subunit epsilon | lmo2528 | 16804566 | Energy Metabolism | ATP Proton Motive Force Interconversion | 14 | 9 | 0.000 | 0.002 | 2 | 0.000 | 0.002 | 2 |
| F0F1 ATP synthase subunit gamma | lmo2530 | 16804568 | Energy Metabolism | ATP Proton Motive Force Interconversion | 11 | 6 | 0.000 | 0.009 | 2 | 0.000 | 0.054 | 2 |
| F0F1 ATP synthase subunit delta | lmo2532 | 16804570 | Energy Metabolism | ATP Proton Motive Force Interconversion | 10 | 3 | 0.003 | 0.027 | 2 | 0.004 | 0.012 | 2 |
|  | lmo2537 | 16804575 | Cell Envelope | Biosynthesis and Degradation of Surface Polysaccharides and Lipopolysaccharides | 6 | 5 | 0.000 | 0.291 | 2 | 0.000 | 0.022 | 2 |
|  | lmo2538 | 16804576 | Purines, Pyrimidines, Nucleosides and Nucleotides | Salvage of Nucleotides and Nucleosides | 16 | 17 | 0.000 | 0.001 | 3 | 0.069 | 0.006 | 3 |
|  | lmo2539 | 16804577 | Multiple Roles | Multiple Roles | 5 | 7 | 0.000 | 0.286 | 2 | 0.101 | 0.254 | 2 |
| 50S ribosomal protein L31 | lmo2548 | 16804586 | Protein Synthesis | Ribosomal Proteins: Synthesis and Modification | 56 | 49 | 0.000 | 0.072 | 6 | 0.000 | 0.291 | 7 |
| hypothetical protein lmo2556 | lmo2556 | 16804594 | Energy Metabolism | Glycolysis/Gluconeogenesis | 43 | 25 | 0.000 | 0.198 | 5 | 0.014 | 0.000 | 4 |
| CTP synthetase | lmo2559 | 16804597 | Purines, Pyrimidines, Nucleosides and Nucleotides | Pyrimidine Ribonucleotide Biosynthesis | 12 | 7 | 0.000 | 0.153 | 2 | 0.069 | 0.052 | 2 |
|  | lmo2560 | 16804598 | Transcription | DNA Dependant RNA Polymerase | 7 | 7 | 0.076 | 0.000 | 2 | 0.000 | 0.001 | 2 |
| arginyl-tRNA synthetase | lmo2561 | 16804599 | Protein Synthesis | tRNA Aminoacylation | 12 | 7 | 0.000 | 0.001 | 2 | 0.066 | 0.000 | 2 |
| hypothetical protein lmo2564 | lmo2564 | 16804602 | Energy Metabolism | Other | 19 | 10 | 0.000 | 0.037 | 3 | 0.000 | 0.017 | 2 |
| peptide/nickel transport system substrate-binding protein | lmo2569 | 16804607 | Function Unknown | Unknown General | 5 | 11 | 0.000 | 0.187 | 2 | 0.000 | 0.000 | 2 |
|  | lmo2577 | 16804615 | Central Intermediary Metabolism | Other | 8 | 8 | 0.000 | 0.000 | 2 | 0.000 | 0.156 | 2 |
|  | lmo2593 | 16804631 | Regulatory Functions | Other | 7 | 5 | 0.010 | 0.089 | 2 | 0.000 | 0.009 | 2 |
|  | lmo2596 | 16804634 | Protein Synthesis | Ribosomal Proteins: Synthesis and Modification | 31 | 32 | 0.000 | 0.008 | 4 | 0.005 | 0.021 | 4 |
| 50S ribosomal protein L13 | lmo2597 | 16804635 | Protein Synthesis | Ribosomal Proteins: Synthesis and Modification | 29 | 10 | 0.010 | 0.089 | 2 | 0.000 | 0.003 | 4 |
|  | lmo2599 | 16804637 | Multiple Roles | Multiple Roles | 5 | 7 | 0.000 | 0.028 | 2 | 0.001 | 0.143 | 2 |
|  | lmo2600 | 16804638 | Transport and Binding Proteins | Unknown Substrate | 5 | 6 | 0.004 | 0.271 | 2 | 0.000 | 0.071 | 2 |
|  | lmo2605 | 16804643 | Protein Synthesis | Ribosomal Proteins: Synthesis and Modification | 7 | 5 | 0.076 | 0.258 | 2 | 0.000 | 0.203 | 2 |
|  | lmo2606 | 16804644 | Transcription | DNA Dependant RNA Polymerase | 9 | 7 | 0.000 | 0.002 | 2 | 0.000 | 0.233 | 2 |
| 30S ribosomal protein S11 | lmo2607 | 16804645 | Protein Synthesis | Ribosomal Proteins: Synthesis and Modification | 6 | 10 | 0.000 | 0.000 | 2 | 0.000 | 0.281 | 2 |
| 30S ribosomal protein S13 | lmo2608 | 16804646 | Protein Synthesis | Ribosomal Proteins: Synthesis and Modification | 53 | 38 | 0.000 | 0.102 | 5 | 0.000 | 0.290 | 6 |
| translation initiation factor IF-1 | lmo2610 | 16804648 | Protein Synthesis | Other | 13 | 22 | 0.076 | 0.071 | 2 | 0.000 | 0.246 | 3 |
| hypothetical protein lmo2611 | lmo2611 | 16804649 | Purines, Pyrimidines, Nucleosides and Nucleotides | Nucleotide and Nucleoside Interconversions | 11 | 5 | 0.000 | 0.146 | 2 | 0.000 | 0.020 | 2 |
| 50S ribosomal protein L15 | lmo2613 | 16804651 | Protein Synthesis | Ribosomal Proteins: Synthesis and Modification | 47 | 16 | 0.000 | 0.000 | 3 | 0.001 | 0.140 | 6 |
|  | lmo2614 | 16804652 | Protein Synthesis | Ribosomal Proteins: Synthesis and Modification | 5 | 7 | 0.069 | 0.003 | 2 | 0.000 | 0.016 | 2 |
| 30S ribosomal protein S5 | lmo2615 | 16804653 | Protein Synthesis | Ribosomal Proteins: Synthesis and Modification | 68 | 57 | 0.000 | 0.003 | 7 | 0.006 | 0.104 | 8 |
| 50S ribosomal protein L18 | lmo2616 | 16804654 | Protein Synthesis | Ribosomal Proteins: Synthesis and Modification | 20 | 7 | 0.000 | 0.000 | 2 | 0.000 | 0.130 | 3 |
| 50S ribosomal protein L6 | lmo2617 | 16804655 | Protein Synthesis | Ribosomal Proteins: Synthesis and Modification | 55 | 21 | 0.000 | 0.023 | 3 | 0.069 | 0.229 | 7 |
| 30S ribosomal protein S8 | lmo2618 | 16804656 | Protein Synthesis | Ribosomal Proteins: Synthesis and Modification | 176 | 18 | 0.000 | 0.224 | 3 | 0.009 | 0.261 | 19 |
| 30S ribosomal protein S14 | lmo2619 | 16804657 | Protein Synthesis | Ribosomal Proteins: Synthesis and Modification | 5 | 10 | 0.000 | 0.187 | 2 | 0.000 | 0.230 | 2 |
| 50S ribosomal protein L5 | lmo2620 | 16804658 | Protein Synthesis | Ribosomal Proteins: Synthesis and Modification | 57 | 34 | 0.000 | 0.002 | 4 | 0.000 | 0.253 | 7 |
|  | lmo2622 | 16804660 | Protein Synthesis | Ribosomal Proteins: Synthesis and Modification | 6 | 5 | 0.076 | 0.005 | 2 | 0.076 | 0.115 | 2 |
| 30S ribosomal protein S17 | lmo2623 | 16804661 | Protein Synthesis | Ribosomal Proteins: Synthesis and Modification | 24 | 6 | 0.000 | 0.016 | 2 | 0.076 | 0.149 | 3 |
| 50S ribosomal protein L29 | lmo2624 | 16804662 | Protein Synthesis | Ribosomal Proteins: Synthesis and Modification | 47 | 16 | 0.000 | 0.000 | 3 | 0.069 | 0.001 | 6 |
|  | lmo2625 | 16804663 | Protein Synthesis | Ribosomal Proteins: Synthesis and Modification | 7 | 5 | 0.076 | 0.000 | 2 | 0.000 | 0.000 | 2 |
| 30S ribosomal protein S3 | lmo2626 | 16804664 | Protein Synthesis | Ribosomal Proteins: Synthesis and Modification | 24 | 19 | 0.000 | 0.002 | 3 | 0.000 | 0.018 | 3 |
| 50S ribosomal protein L22 | lmo2627 | 16804665 | Protein Synthesis | Ribosomal Proteins: Synthesis and Modification | 120 | 46 | 0.076 | 0.001 | 6 | 0.000 | 0.088 | 13 |
| 30S ribosomal protein S19 | lmo2628 | 16804666 | Protein Synthesis | Ribosomal Proteins: Synthesis and Modification | 24 | 15 | 0.069 | 0.099 | 3 | 0.000 | 0.003 | 3 |
| 50S ribosomal protein L2 | lmo2629 | 16804667 | Protein Synthesis | Ribosomal Proteins: Synthesis and Modification | 21 | 14 | 0.069 | 0.186 | 2 | 0.000 | 0.000 | 3 |
| 50S ribosomal protein L23 | lmo2630 | 16804668 | Protein Synthesis | Ribosomal Proteins: Synthesis and Modification | 110 | 33 | 0.000 | 0.017 | 4 | 0.000 | 0.084 | 12 |
|  | lmo2631 | 16804669 | Protein Synthesis | Ribosomal Proteins: Synthesis and Modification | 19 | 18 | 0.000 | 0.160 | 3 | 0.000 | 0.012 | 3 |
| 50S ribosomal protein L3 | lmo2632 | 16804670 | Protein Synthesis | Ribosomal Proteins: Synthesis and Modification | 22 | 10 | 0.069 | 0.152 | 2 | 0.000 | 0.052 | 3 |
| RS10_LISMO | lmo2633 | 16804671 | Protein Synthesis | Ribosomal Proteins: Synthesis and Modification | 46 | 12 | 0.076 | 0.015 | 2 | 0.000 | 0.185 | 6 |
| hypothetical protein lmo2637 | lmo2637 | 16804675 | Cell Envelope | Other | 10 | 23 | 0.076 | 0.149 | 2 | 0.101 | 0.018 | 3 |
| hypothetical protein lmo2638 | lmo2638 | 16804676 | Central Intermediary Metabolism | Other | 41 | 19 | 0.000 | 0.013 | 3 | 0.000 | 0.003 | 5 |
| elongation factor Tu | lmo2653 | 16804690 | Protein Synthesis | Other | 198 | 90 | 0.000 | 0.001 | 21 | 0.000 | 0.009 | 10 |
| elongation factor G | lmo2654 | 16804691 | Protein Synthesis | Other | 57 | 37 | 0.000 | 0.000 | 5 | 0.064 | 0.250 | 7 |
| 30S ribosomal protein S7 | lmo2655 | 16804692 | Protein Synthesis | Ribosomal Proteins: Synthesis and Modification | 15 | 8 | 0.000 | 0.000 | 2 | 0.069 | 0.058 | 3 |
| hypothetical protein lmo2666 | lmo2666 | 16804703 | Multiple Roles | Multiple Roles | 8 | 13 | 0.000 | 0.003 | 2 | 0.000 | 0.000 | 2 |
| hypothetical protein lmo2683 | lmo2683 | 16804720 | Multiple Roles | Multiple Roles | 15 | 73 | 0.001 | 0.173 | 3 | 0.101 | 0.248 | 8 |
| hypothetical protein lmo2685 | lmo2685 | 16804722 | Multiple Roles | Multiple Roles | 12 | 34 | 0.000 | 0.072 | 2 | 0.000 | 0.000 | 4 |
| hypothetical protein | lmo2692 | 16804729 | Function Unknown | Unknown General | 5 | 12 | 0.000 | 0.002 | 2 | 0.000 | 0.241 | 2 |
|  | lmo2695 | 16804732 | Multiple Roles | Multiple Roles | 5 | 6 | 0.000 | 0.001 | 2 | 0.000 | 0.000 | 2 |
| hypothetical protein lmo2696 | lmo2696 | 16804733 | Multiple Roles | Multiple Roles | 9 | 21 | 0.000 | 0.008 | 2 | 0.000 | 0.001 | 3 |
| hypothetical protein lmo2700 | lmo2700 | 16804737 | Central Intermediary Metabolism | Other | 13 | 5 | 0.000 | 0.001 | 2 | 0.000 | 0.002 | 2 |
| hypothetical protein | lmo2703 | 16804740 | Function Unknown | Unknown General | 5 | 17 | 0.076 | 0.005 | 2 | 0.101 | 0.017 | 3 |
| hypothetical protein lmo2707 | lmo2707 | 16804744 | Function Unknown | Unknown General | 49 | 102 | 0.000 | 0.016 | 6 | 0.000 | 0.055 | 11 |
| hypothetical protein lmo2709 | lmo2709 | 16804746 | Function Unknown | Unknown General | 24 | 5 | 0.000 | 0.000 | 3 | 0.000 | 0.013 | 2 |
|  | lmo2728 | 16804765 | Regulatory Functions | Other | 5 | 6 | 0.076 | 0.258 | 2 | 0.000 | 0.100 | 2 |
|  | lmo2743 | 16804780 | Energy Metabolism | Pentose Phosphate pathway | 6 | 9 | 0.076 | 0.015 | 2 | 0.101 | 0.032 | 2 |
|  | lmo2747 | 16804784 | Protein Synthesis | tRNA Aminoacylation | 5 | 6 | 0.000 | 0.125 | 2 | 0.006 | 0.020 | 2 |
|  | lmo2754 | 16804791 | Cell Envelope | Biosynthesis and Degradation of Murein Sacculus and Peptidoglycan | 5 | 6 | 0.069 | 0.229 | 2 | 0.092 | 0.207 | 2 |
| IMP dehydrogenase | lmo2758 | 16804795 | Purines, Pyrimidines, Nucleosides and Nucleotides | Purine Ribonucleotide Biosynthesis | 22 | 32 | 0.000 | 0.054 | 3 | 0.000 | 0.257 | 4 |
|  | lmo2785 | 16804822 | Function Unknown | Unknown General | 7 | 7 | 0.076 | 0.000 | 2 | 0.101 | 0.031 | 2 |
|  | lmo2790 | 16804827 | Cellular Processes | Cell Division | 7 | 6 | 0.000 | 0.001 | 2 | 0.000 | 0.021 | 2 |
|  | lmo2792 | 16804829 | Regulatory Functions | DNA Interactions | 6 | 9 | 0.000 | 0.000 | 2 | 0.069 | 0.000 | 2 |
|  | lmo2811 | 16804848 | Protein Synthesis | Translation Factors | 9 | 8 | 0.000 | 0.001 | 2 | 0.000 | 0.036 | 2 |
|  | lmo2828 | 16804865 | Function Unknown | Unknown General | 7 | 5 | 0.000 | 0.002 | 2 | 0.000 | 0.080 | 2 |
| hypothetical protein lmo2853 | lmo2853 | 16804890 | Function Unknown | Unknown General | 7 | 19 | 0.076 | 0.001 | 2 | 0.101 | 0.000 | 3 |
